# Correlative light and soft X-ray tomography of in situ mesoscale heterochromatin structure in intact cells

**DOI:** 10.1038/s41598-024-77361-2

**Published:** 2024-11-12

**Authors:** Rajshikhar Gupta, Yagyik Goswami, Luezhen Yuan, Bibhas Roy, Eva Pereiro, GV Shivashankar

**Affiliations:** 1 Laboratory of Nanoscale Biology, Paul Scherrer Institut, Villigen, Aargau Switzerland; 2 Department of Health Sciences and Technology, ETH Zürich, Zürich, Switzerland; 3 ALBA Synchrotron Light Source, Cerdanyola del Vallés, Barcelona Spain; 4 Department of Biological Sciences, BITS Pilani Hyderabad Campus, Secunderabad, India

**Keywords:** X-ray tomography, Biopolymers in vivo, Nucleus

## Abstract

Heterochromatin organization is critical to many genome-related programs including transcriptional silencing and DNA repair. While super-resolution imaging, electron microscopy, and multiomics methods have provided indirect insights into the heterochromatin organization, a direct measurement of mesoscale heterochromatin ultrastructure is still missing. We use a combination of correlative light microscopy and cryo-soft X-ray tomography (CLXT) to analyze heterochromatin organization in the intact hydrated state of human mammary fibroblast cells. Our analysis reveals that the heterochromatin ultra-structure has a typical mean domain size of approximately 80 nm and a mean separation of approximately 120 nm between domains. Functional perturbations yield further insights into the molecular density and alterations in the mesoscale organization of the heterochromatin regions. Furthermore, our polymer simulations provide a mechanistic basis for the experimentally observed size and separation distributions of the mesoscale chromatin domains. Collectively, our results provide direct, label-free observation of heterochromatin organization in the intact hydrated state of cells.

**Supplementary Information:**

The online version contains supplementary material available at 10.1038/s41598-024-77361-2.

## Introduction

Heterochromatin is a structural feature of eukaryotic cells and comprises a dense organization of highly conserved, repeated sequences of chromosomal DNA. Heterochromatin spatial organization plays a crucial role in regulating important functions such as transcriptional silencing, maintaining genome stability, DNA repair, and ensuring proper chromosome segregation during cell division^1^. Various multi-omics studies have shown that cohesive interactions between epigenetic marks are crucial to the formation of heterochromatin structure^2^. Importantly, chromosome conformation capture methods have revealed that the genome is organized into topologically associated domains^3,4^. Recent super-resolution imaging shows highly heterogenous length scales associated with the epigenetic modifications to condensed chromatin regions, ranging from 50 to 100 nm^5,6^. These results indicate varying levels of spatial compaction in the native structure of heterochromatin. In addition, studies of heterochromatin dynamics using live cell fluorescent imaging methods reveal the existence of spatiotemporally segregated stable domains of condensed states within heterochromatin regions^7–9^. Such local and global compaction of native heterochromatin structure is believed to play an essential functional role during the development and maintenance of cellular homeostasis^10,11^. While these studies have provided important information about heterochromatin spatial organization, insights into the in-situ mesoscale heterochromatin structure in the intact hydrated state of cells is still far from clear. Moreover, the theoretical basis for such heterochromatin organization is not well understood.

In this paper, we take advantage of CLXT to directly access the architecture of the native heterochromatin structure in the intact hydrated state of cells. Soft X-ray imaging methods have a unique advantage over conventional super-resolution and cryo electron microscopy methods as it allows for label-free visualization of intact and hydrated biospecimen at the resolution of 30–50 nm in situ^10,12^. Correlating light microscopy images with soft X-ray tomography has enabled direct visualization of morphological changes in cellular organelles with fast acquisition times ($<20$ min/cell)^13–15^. From a theoretical perspective, the global spatial organization of chromatin has elicited long-standing interest, with immense insight obtained from polymer physics models^16^. Such modeling approaches have also elucidated the basis for the formation of highly complex structural features such as extruded loops and topologically associated domains (TADs)^17,18^. However, the spatial organization of dense heterochromatin mesoscale domains at the fine-grained length scale discussed in this work remains relatively unexplored with such modeling approaches. We provide a direct measurement of the mesoscale architecture of label-free heterochromatin and find spatially segregated mesoscale domains in the native structure of heterochromatin in intact cells. We find that the mesoscale heterochromatin structure has spatial and structural heterogeneity with length scales of a mean size of $\approx 80$ nm, with variability of 35 nm–250 nm. We show that the various histone modifications tightly regulate such mesoscale heterochromatin organization. We then perform Brownian dynamics simulations in the framework of a polymer model^19^ to investigate the effect of the affinity between distinct chromatin regions on the mesoscale organization of higher-order chromatin structure. Our simulations produce structures in agreement with those observed through CLXT, in particular, the size and separation distributions of the mesoscale domains. Collectively, our CLXT imaging and polymer simulations provide direct evidence of mesoscale chromatin domains as well as a possible mechanistic basis for their organization.

## Results

### CLXT imaging reveals heterochromatin ultrastructure

**Fig. 1:**
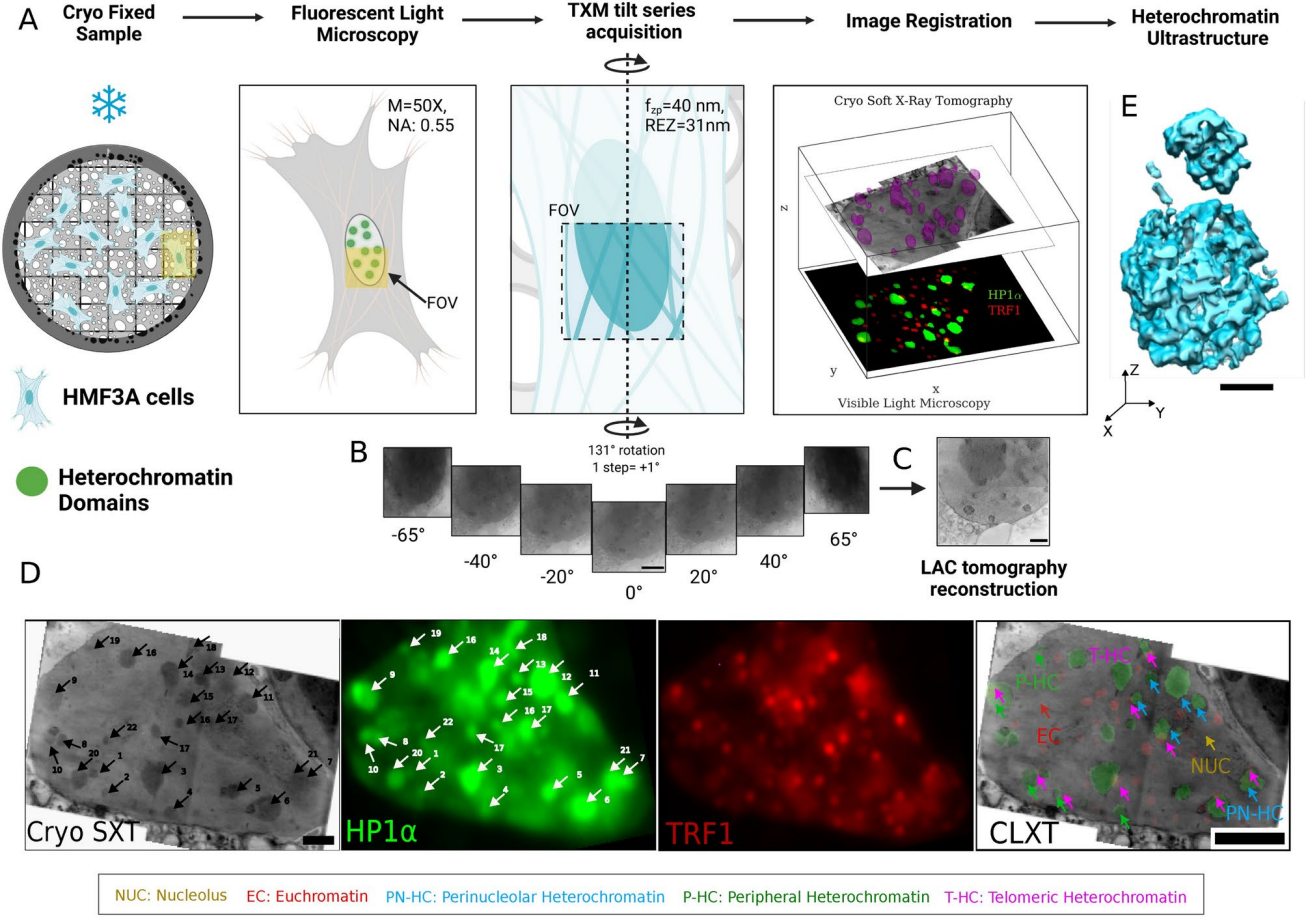
CLXT reveals heterochromatin ultrastructure. (**A**) Schematic overview of CLXT imaging workflow for the visualization of HMF3A heterochromatin ultrastructure generated from multiple fields of view (FOV) of the subnuclear regions. (**B**) TXM absorption projections from the FOV from the − 65 to 65 degrees of rotation with the visible nucleolus, nuclear boundary, and heterochromatin regions. (**C**) LAC tomography reconstruction of the tilt series of TXM absorption projection of subnuclear FOV. (**D**) Maximum Z projection of stitched LAC tomogram of multiple FOVs, corresponding visible light microscopy of HP1$\alpha$-GFP and TRF1-DsRed tagged HMF3A nucleus, numbers and arrow labels corresponding to each heterochromatin node in cryo SXT and CLXT virtual slices. Correlated and merged visualization of euchromatin, nucleolus, telomeric, perinucleolar, and peripheral heterochromatin regions in the intact, hydrated nucleus as indicated in the legend by color-coded arrows. (**E**) 3D visualization of spatially connected mesoscale domains of condensed chromatin ultra-structure in situ. Scale Bars: (**B**) 4 $\upmu$m (**C**) 2 $\upmu$m (**D**) 5 $\upmu$m (**E**) 1 $\upmu$m.

We developed a CLXT imaging workflow wherein we use the functional landmarks associated with heterochromatin to co-localize them with the spatial linear absorption coefficient distribution (LAC) (see “Methods”) (Fig. 1A). In order to visualize the label-free native heterochromatin organization, we use the adherent Human Mammary Fibroblasts (HMF3A) cell line as they present visually characterizable spherical, dense heterochromatin regions at perinucleolar regions, and at the nuclear boundaries (Figs. 1A, S1A, S8A,B,C, S21, S22). Given that genome organization and regulation is highly sensitive to cellular geometry^20,21^, we ensured that the adherent HMF3A fibroblast cells were imaged in their native cell geometry i.e. unconfined, adhered, and elongated. To this end, we obtain the fluorescent images of cryo-fixed, adherent HMF3A cells labeled with telomere (dsRed TRF1) and heterochromatin (HP1$\alpha$ GFP) attached to the carbon-coated gold grids as shown in Fig. 1A. For the same cells, we obtained the tilt series of Transmission X-Ray Microscopy (TXM) absorption projections of subnuclear regions over the angular range of 131 $^{\circ }$ (Fig. 1B), optimal for imaging adherent cells^13,22^. We then reconstruct the deconvolved and aligned absorption projections of subnuclear regions using the algebraic reconstruction approach (Fig. 1C and Fig. S2A in the Supplementary Materials). We then validate that the dense spherical mesoscale domains from the SXT colocalize with HP1$\alpha$ rich heterochromatin regions (see “Methods”) by aligning the light microscopy and TXM absorption projections (Fig. 1D and Fig. S2D in the Supplementary Materials). Our tilt series reconstruction of the subnuclear Field of View (FOV) provides a spatial resolution of 31 nm (Fig. S1B,G in the Supplementary Materials) half-pitch. In the LAC-reconstructed SXT, we could also visualize other organelles within the nuclear membrane and cytoplasm (Fig S1I,K in the Supplementary Materials) and the inner and outer nuclear membrane (Fig. S1E–G in the Supplementary Materials). Our feature-based alignment method provides us with a 2D alignment accuracy of 660 nm for features of 1–2 $\upmu$m (Fig. S1C in the Supplementary Materials). Our CLXT imaging workflow produced similar alignment accuracy across sample preparation methods (Fig. S1D in the Supplementary Materials). In Fig. 1E, we show the mesoscale ultrastructure of heterochromatin in the intact and hydrated cells using the CLXT imaging method after thresholding the spatial LAC distribution (see “Methods”). Additionally we also performed CLXT on other cell lines as demonstrated in Fig. S24A–C. It can be seen that there is a notable visual correlation between the high-intensity bright regions stained with Hoechst in cryo-light microscopy and the dense, dark regions with higher linear absorption coefficients (LAC) in the cryo-SXT virtual slices even in the relatively intricate chromatin organization. Taken together, these images provide a label-free visualization of heterochromatin organization^7,8^.

### Mesoscale heterochromatin structure has spatial and structural heterogeneity

**Fig. 2:**
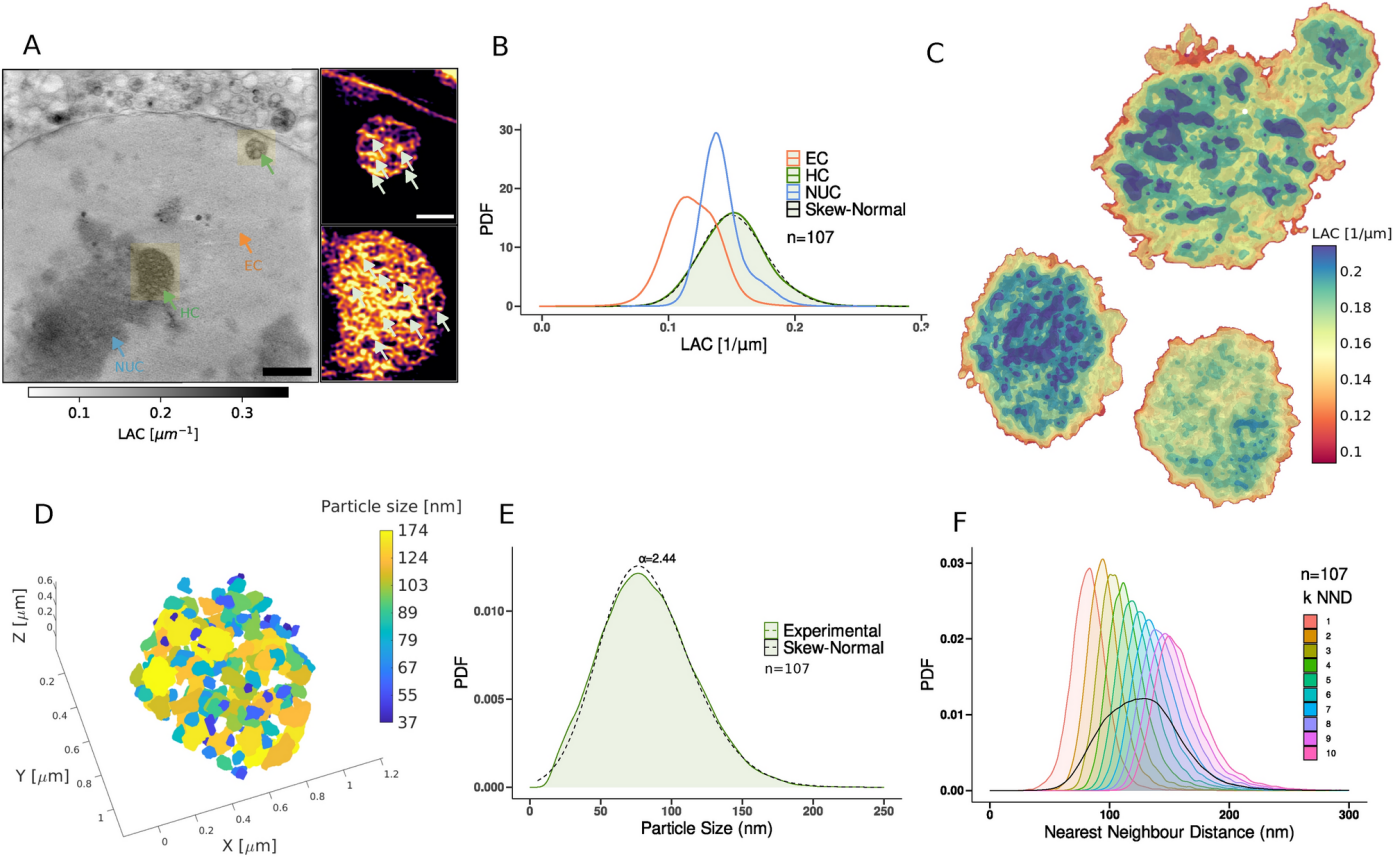
Mesoscale heterochromatin 3D ultrastructure has spatial heterogeneity. *NUC* nucleolus, *EC* euchromatin, *HC* heterochromatin, Numbers in (**A**) and (**C**) indicate corresponding HC domains. (**A**) Representative virtual Z slice from FOV of subnuclear Cryo-SXTs, indicating nucleolus, euchromatin, and heterochromatin marked with color-coded arrows. Inset: Yellow Regions of Interest (ROI) are zoomed in and color-mapped with a local LAC range, arrows indicating the characteristic connected clusters of dense domains in HMF3A$^{WT}$ cells. (**B**) The distribution of LAC of heterochromatin in comparison with the nucleolus, and euchromatin across all the measurements in HMF3A$^{WT}$ cells, n = 107 indicates the number of individual heterochromatin regions. (**C**) 3D visualization of LAC distribution in heterochromatin regions showing regions with LAC values ranging from 0.1 $\upmu$m$^{-1}-$ 0.25 $\upmu$m$^{-1}$. LAC is mapped with a linear piecewise opacity map in HMF3A$^{WT}$ cells. (**D**) 3D visualization of heterochromatin mesoscale domain segmented from the heterochromatin regions mapped with a color bar indicating the particle sizes as effective diameter. (**E**) Size distribution of heterochromatin mesoscale domains fitted to skew-normal distribution with skewness $\alpha$. (**F**) Distribution of k-Nearest neighbor distances for k = 1–10 color-coded according to the color bar, along with cumulative distribution indicated in black. The total number of cells analysed, $n_{cells}=7$. Scale Bar: (**A**) 2 $\upmu$m, Inset: 0.5 $\upmu$m.

To quantitatively assess the obtained ultrastructure of heterochromatin organization (Fig. 2A, Fig. S10), we characterize the volumetric packing densities using the reconstructed LAC of the soft X-ray tomograms. We find that the LAC values of heterochromatin region range from 0.1 to 0.27 $\upmu$m$^{-1}$, significantly higher than euchromatin regions with LAC values ranging from 0.05 to 0.22 $\upmu$m$^{-1}$ (Fig. 2B). We find that the density distribution of heterochromatin domains (0.15 to 0.27 $\upmu$m$^{-1}$), is surrounded by regions of low density (0.1 to 0.15 $\upmu$m$^{-1}$) as shown in Fig. 2C. The characteristic low and high-density domain organization is not due to the over-expression of HP1$\alpha$ and is present in both HMF3A$^{WT}$ and HMF3A$^{HP1\alpha -GFP}$ cell lines (Fig. S8D, S8E in the Supplementary Materials). We further assessed the maximum possible radiation dose received during the tilt-series acquisition as shown in Fig. S9A using the method suggested in previous studies and found it well below the recommended limit of $10^9$ Gy^23^. Additionally, we captured the TXM projection before and after each tomogram acquisition to compare the images and ensure no visible damage occurred (Fig. S23). This implies that the observed mesoscale heterochromatin domains were not artifacts of radiation damage. We also note that heterochromatin organization is spatially heterogenous within the same nucleus controlled for their size (Fig. 2C and Fig. S3A, S11–S14 in the Supplementary Materials). We segment the individual dense domains to characterize the structural features of the dual-phase heterochromatin organization (see “Methods”). We find that the average domain size of densely connected regions is of $\approx 80$ nm as effective diameter, and ranges between 30–250 nm (Fig. 2E). This observation is in agreement with previous super-resolution studies which report a large heterogeneity in the domain sizes using fluorescent labeling of functional histone modifications^5,11^. In order to characterize the internal architecture of the condensed heterochromatin we then calculate the domain-to-domain K-nearest neighbor distances. We find that the heterochromatin is sparsely packed with an average domain separation of $\approx ~120$ nm (Fig. 2F). Collectively our analysis suggests that the heterochromatin ultrastructure is organized into spatially connected mesoscale domains and is heterogeneous within the intact hydrated cell nucleus.

### Mesoscale heterochromatin organization is regulated by histone modifications

**Fig. 3:**
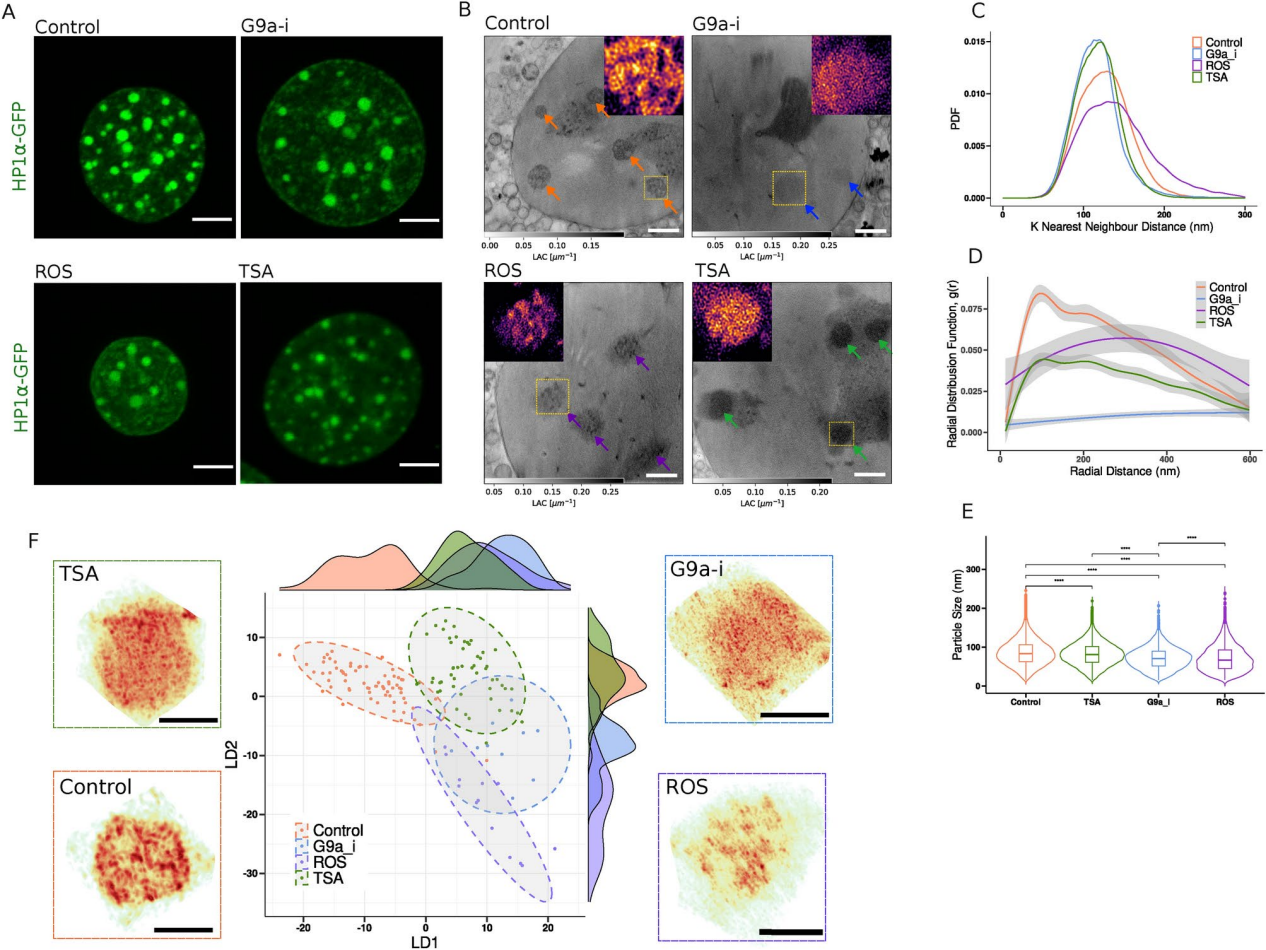
Histone modifications regulate 3D mesoscale organization of heterochromatin. (**A**) Visible light microscopy images of HP1$\alpha$-GFP tagged live HMF3A nuclei showing the microscopic effect of TSA, G9a inhibition, and ROS stimulation against control. (**B**) Cryo SXT virtual slice of treatment conditions shows the mesoscale changes in heterochromatin organization upon TSA, G9a inhibition, and ROS stimulation against control in HMF3A$^{WT}$ cells. Color-coded arrows indicate the heterochromatin regions at the perinucleolar and peripheral heterochromatin. Inset: The yellow region of interest is zoomed in and color-mapped with a local intensity range. (**C**–**E**) Cumulative K nearest neighbor distances (K = 1 to 10), the pair-correlation function *g*(*r*), particle size differences in mesoscale domains upon TSA, G9a inhibition, and ROS stimulation against control. (**F**) Linear Discriminant (LD) Analysis plot of multi-parametric descriptive features of heterochromatin organization showing conditional clustering, indicated by 95% confidence level for a multivariate t-distribution. *ROI* Representative volumes, representing the spatial distribution of LAC, warmer colors representing greater LAC, and cooler colors representing vice versa. Scale Bars: (**A**) 5 $\upmu$m, (**B**) 2 $\upmu$m. (**C**) 1 $\upmu$m (**F**) 1 $\upmu$m (**E**) $p<0.0001$ (Mann Whitney test) The total number of cells analysed, $n_{control} = 7, n_{TSA}=7, n_{G9a}=4, n_{ROS}=4$.

The observed dense heterochromatin domains could be a result of various histone modifications. We therefore reason that alterations to these modifications should modulate the architecture of the heterochromatin domains. Several studies have shown that treatment with Trichostatin-A (TSA), a histone deacetylase inhibitor, leads to global acetylation and heterochromatin decondensation^24,25^. Consistent with this, TSA treatment resulted in marked disruptions in the 3D mesoscale organization of heterochromatin ultrastructure in HMF3A$^{WT}$ cells (Fig. 3B and Fig. S3B, S11–20 in the Supplementary Materials). We also find a significant decrease in the size and compaction length scales of the dense mesoscale domains (Fig. 3C–E, S11–S20). Recent studies have shown that inhibition of G9a, a histone methyl transferase, is associated with altered chromatin accessibility and loss of heterochromatin^26,27^. We, therefore, treated the cells with a G9a inhibitor and analyzed its effect on heterochromatin 3D organization. G9a inhibition resulted in diffused HP1$\alpha$-enriched heterochromatin regions in visible light microscopy and with LAC value closer to euchromatin as shown in cryo SXT virtual slice (Fig. 3A, B, Fig. S3C, and S25A in the Supplementary Materials) and quantified in (Fig. S3F in the Supplementary Materials). We also observe a decrease in particle size, disordered local and global mesoscale domain organization, and a decrease in compaction length scale in HMF3A$^{WT}$ cells (Fig. 3C–E,  S11–S20). We next tested the role of DNA strand breaks on the ultrastructure of the heterochromatin domain organization. PPKO has been shown to increase reactive oxygen species (ROS) activity and thus induce DNA strand breaks^28,29^. After PPKO treatment, we observe rounding and shrinkage of nuclear volume in both light microscopy and cryo-SXT (Fig. 3A, B, Fig. S3D). Spatial LAC distribution shows the decrease in the prominence of heterochromatin domain boundaries at the perinucleolar regions with clustered mesoscale domains of larger compaction length scales in HMF3A$^{WT}$ cells (Fig. 3C–E). Based on these observations we conclude that the 3D organization of mesoscale domains is highly sensitive to epigenetic changes and chromatin fiber integrity.

We created a list of both heterochromatin-wide and mesoscale domain-level features to characterize the overlap of inter-class similarity and intra-class variability among each functional perturbation (see “Methods”, Fig. S4A,B in the Supplementary Materials). Linear Discriminant Analysis (LDA) of descriptive features of heterochromatin organization distinguishes the mesoscale morphological, organizational, and spatial changes in LAC values across various treatment conditions with an average accuracy of $98\%$ in heterochromatin wide and $68\%$ accuracy in mesoscale domain level organization (Fig. S5A,D in the Supplementary Materials). At the population level, the heterochromatin regions from different treatment conditions form distinct clusters indicating that the experimental data collected across multiple trials are highly reproducible (Fig. 3F).

### Polymer modeling reveals route to heterochromatin ultrastructure formation

**Fig. 4:**
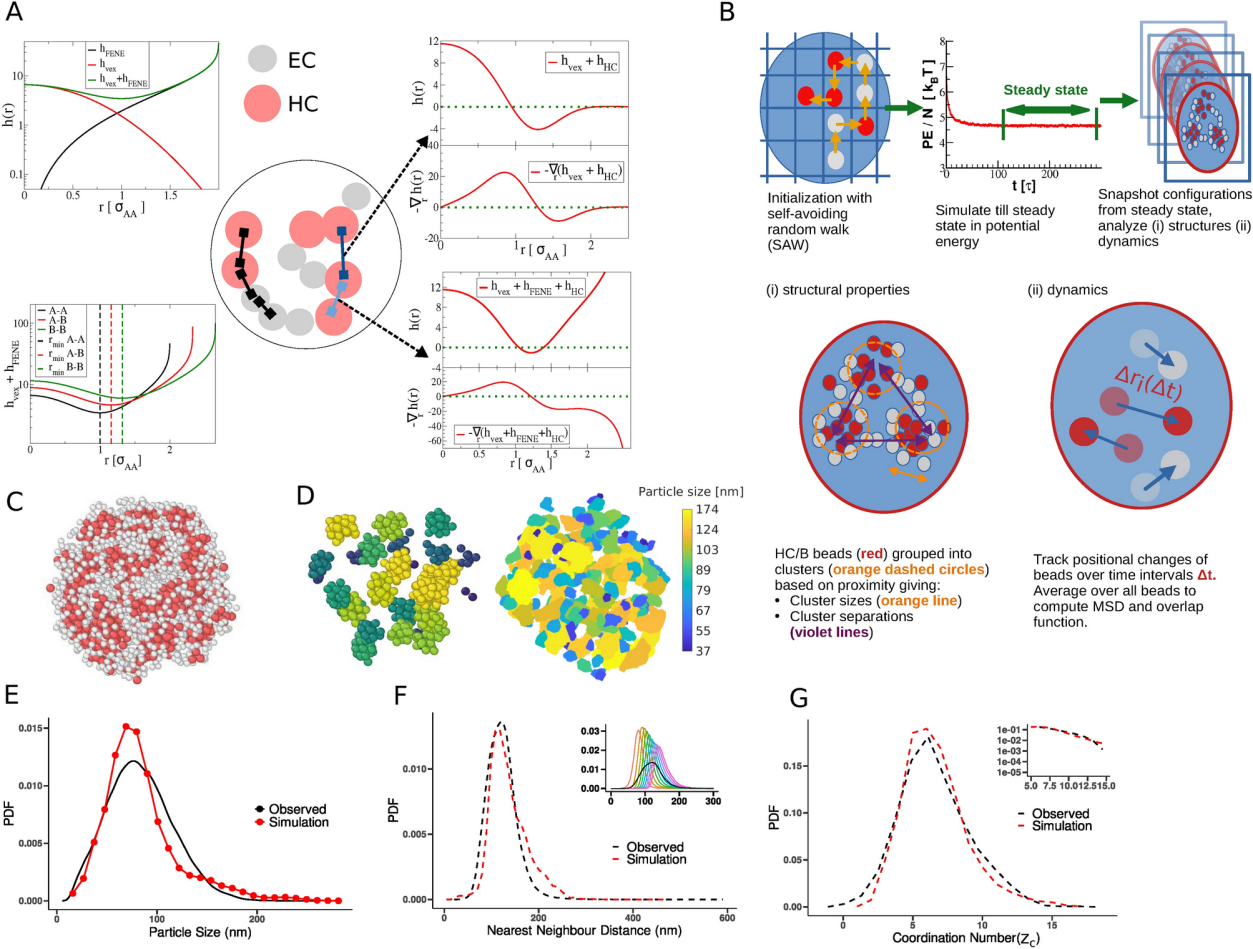
Polymer modeling reveals route to heterochromatin ultrastructure formation. (**A**) Schematic representation of the different pairwise interactions. The soft core repulsion and entropic spring combine to produce a net interaction between all beads that are neighbors on the chain as in the bottom left. Pairs of HC/*B* beads (red) that are neighbors along the chain interact with an attraction as well as the soft core and spring (bottom right) and other non-neighbor pairs of HC/*B* beads do not have the entropic spring interaction. (**B**) Schematic depiction of the simulation workflow. Snapshots of bead coordinates from each trajectory are stored periodically and used to analyse the clustering of the HC/*B* beads, as well as to compute dynamical properties such as the mean squared displacement. (**C**) Shows a typical snapshot from the polymer simulation with clustered HC/*B* beads (red) and EC/*A* beads (white) in the interstitial spaces. (**D**) Identifying the clusters of HC/*B* beads and applying a color scale proportional to size (blue—small; yellow—large) allows a visual comparison with the spatial organization of segmented mesoscale domains from CLXT. The domain size distributions from simulation and experiment are shown in (**E**), the distribution of nearest neighbor separations in (**F**), and the distribution of the coordination number, $z_c$, in (**G**).

Polymer physics approaches have generated tremendous insight into the 3D organization of chromatin^30,31^. A recent study highlighted that the compartmentalization of chromatin into active *A* and inactive *B* regions can be understood to result from a combination of heterochromatin affinity and the activity of the topoisomerase enzyme^19^. Here, we utilize this framework to elucidate the formation of the dense mesoscale domains as evident in the molecular density maps generated through the CLXT imaging (see Fig. 1). We perform Brownian dynamics simulations of a polymer chain in a confined spherical volume (see “Methods”) and study the resultant clustering of heterochromatin regions. Beads are either euchromatin (EC/*A*) or heterochromatin (HC/*B*), representing groups of nucleosomes having either open/active marks, or inactive marks on the histone tails respectively. Figure 4A summarises the interactions between the EC/*A* beads (white beads in Fig. 4A–C) representing contiguous sequences of nucleosomes enriched in “active” histone modifications such as H3K9Ac^5^ and HC/*B* beads representing contiguous sequences of nucleosomes enriched in “inactive/repressive” H3K9me3/H3K27me3 modifications^32^. Heterochromatin affinity between the HC/*B* beads (red beads in Fig. 4A–C), understood to arise through an interplay of the activity of HP1$\alpha$, methyl transferases, and methylated histones^33,34^ is modeled along the lines in^19^. We first equilibrate the polymer chain in confinement, monitoring the potential energy over time (see Fig. S6G in the Supplementary Materials), which attains a constant value at a steady state. A schematic summary of the simulation workflow is presented in Fig. 4B. Interestingly, one observes a striking visual correspondence between the mesoscale domains observed with CLXT imaging and the mesoscale clusters of HC/*B* beads in simulations in Fig. 4D. A comparison of cluster sizes in Fig. 4E (see “Methods” for definition) with the mesoscale domain sizes as resolved in Fig. 2 is found to be in agreement for strong heterochromatin affinity, highlighting the utility of this approach in demonstrating a potential mechanism for heterochromatin ultrastructure formation. In Fig. S6A in the Supplementary Materials, we observe that the mean squared displacement, $\langle r^2(t) \rangle$, scales as $t^{1/2}$, characteristic of polymers^35^, with a separation between the diffusivities of the EC/*A* and HC/*B* beads in the regime of heterochromatin affinity ($\epsilon _{HC}$) where clustering occurs. The corresponding overlap functions in Fig. S6B in the Supplementary Materials—the fraction of beads to have moved less than their diameter in a given time interval (see “Methods”)—shows a similar separation, with HC/*B* beads relatively immobile as a result of being clustered, while EC/*A* beads move comparatively more on the same timescale. Fits to stretched exponential functions give an estimate of the times over which an HC/*B* cluster can be expected to be persistent, which is of the order of 100 ms. Analogously to the comparison of cluster/domain sizes, the nearest neighbor distances between clusters/domains, shown in Fig. 4F also have strong correspondence with those observed in control cells. In Fig. 4G, we analyze the distribution of the number of neighbors for each cluster, termed the coordination number, $z_c$. A similar analysis is performed on the segmented clusters observed through CLXT imaging where nearest neighbors are defined equivalently but without explicit connectivity information. The theoretical comparison of the distributions of the coordination number, $z_c$, is strikingly similar to the experiments (see Fig. 4G).

Our theoretical framework suggests that cluster formation is driven by a combination of (i) the affinity of heterochromatin (clustered HC/*B* bead) regions represented by the attraction term (see “Methods”), and (ii), an entropic expulsion of euchromatic (EC/*A* bead) regions to the surface of the domains, resulting from the highly dynamic EC/*A*-type beads seeking to maximize their entropy, i.e., available volume, compared to relatively condensed and immobile HC/*B*-bead rich regions.

Functional perturbations to histone modifications that disrupt the biochemical activity drastically alter the spatial organization and characteristics of the heterochromatin mesoscale domains, as summarised in Fig. 3. Simulations where we consider a lowering of the heterochromatin affinity display strikingly similar trends in the reduction of the typical cluster sizes, shown in Fig. S7 in the Supplementary Materials. Note that in the regime of low heterochromatin affinity, the steady state is achieved more rapidly with a higher value of the potential energy per bead (see Fig. S6G in the Supplementary Materials), whereas at higher values $\epsilon _{HC}\ge 3$, the potential energy relaxes to steady state logarithmically. Figure S6H and Fig. S6I show the mean squared displacements of EC/*A* and HC/*B* type beads respectively, for different regimes of heterochromatin affinity. At low $\epsilon _{HC}$, the overall dynamics is higher, without any separation in the mobility of EC/*A* and HC/*B* beads. As $\epsilon _{HC}$ is increased, a separation appears between the MSD of EC/*A* beads and that of HC/*B* beads, with HC/*B* beads slowing down to a greater degree. We find through our simulations, that a heterochromatin affinity value of $\epsilon _{HC}=3$ corresponds to the control condition (Fig. 3 and Fig. S7). A decrease in this affinity to $\epsilon _{HC}=2.5$ results in the disrupted heterochromatin mesoscale domain organization, mimicking the effect of the G9a inhibitor. A further decrease to $\epsilon _{HC}=2$, results in the spatial organization mimicking the effect of increased ROS activity.

## Discussion

The unique advantages of CLXT imaging enable us to capture the ultrastructure and spatial density distribution of heterochromatin. We discuss this in the context of earlier studies that showed the existence of heterogenous length scales associated with TAD domains ranging from a few 100 kbp to a few Mbp using chromatin conformation capture methods^3,4^. Moreover, super-resolution studies have also found a diverse range of sizes in heterochromatin-specific histone modifications ranging from 50 nm to 100 nm^5^. A recent super-resolution study also showed via indirect fluorescent labels to Histone H3 lysine 9 trimethylation the existence of discrete domains of similar sizes within heterochromatin regions with marked changes in the global organization and in packing length scales during cancer progression^11^. Since the above studies were indirect, we now visualize this spatial packing heterogeneity in the native structure of heterochromatin using CLXT imaging in label-free, hydrated, and intact cells. We also want to highlight that the heterochromatin organization is cell type and cell state specific. The round spherical domains of heterochromatin observed in our study were also present in other cell types such as NIH3T3^7,36^. The characteristic chromatin organization found in the HMF3A cell line has also been shown using confocal imaging in previous studies, in particular^37,38^. We observe spherical heterochromatin regions in HMF3A cells in all our control and over-expression conditions, both in fixed and in live cell imaging as shown in Fig. S8A–C. Taken together, we find that the global nuclear localization of heterochromatin regions as observed in our study was in line with the past literature^7,36–38^. Moreover, we show that heterochromatin ultrastructure forms condensed and spatially connected clusters of mesoscale domains of size $\approx ~81$ nm ranging from 30 nm to 250 nm of effective diameter with the average spatial separation of $\approx ~120$ nm. Interestingly at the half-pitch resolution of 31 nm, within the same nucleus, we have observed that the heterochromatin regions have considerable spatial heterogeneity in their molecular density distribution and spatial arrangement of the condensed domains. This is in line with the recent observations that found the existence of dynamic and partially looped TADs^39^ and present novel implications on the physical properties of such heterochromatin arrangement. Several studies have observed disruption in the spatial segregation and global 3D organization of chromatin upon global acetylation using super-resolution light and electron microscopy studies^8,40,41^. Upon treatment with TSA, the ultrastructure of heterochromatin is altered suggesting that the chromatin exists in spatially segregated condensed states. G9a is a key methyltransferase regulating methylation states of histone H3 lysine 9 and its inhibition leads to an increase in chromatin associability and the loss of heterochromatin with marked changes in its morphology as observed through ATAC seq and electron microscopy^26,27^. Consistent with this, we observe the dissolution of heterochromatin mesoscale domains, disruption in heterochromatin morphology, and euchromatin-like molecular density of heterochromatin regions upon G9a inhibition using cryo SXT. The effect of ROS stimulation is extensively studied in the context of oxidative stress-implicated DNA damage by the introduction of double-strand breaks (DSB) in the chromatin^28,29^. This is accompanied by heterochromatin to euchromatin conversion and increased accessibility of DNA to initiate DNA repair response^42^. Exogenous stimulation of ROS indeed shows less prominent heterochromatin boundaries and the overall loss of heterochromatin regions using cryo SXT.

In order to gain mechanistic insights, we present a phenomenological polymer model of distinct chromatin domains where ingredients of the experimental measurements were incorporated into a framework of specified interaction rules. In this framework, we consider two kinds of beads, EC/*A* and HC/*B*, where the HC/*B* beads represent contiguous regions of chromatin with histone modifications particular to heterochromatin. All beads have a soft repulsive core while the HC/*B* beads have an additional affinity interaction that induces their clustering. Neighboring beads along the length of the polymer additionally interact with an entropic spring, while the entire simulation is carried out within a spherical cavity of constant volume with repulsive walls. These simulations provided a direct comparison with the experimentally observed domain size distributions and domain organization in cells in the intact, hydrated state. While various polymer physics models have been developed to study chromatin in general, the direct correspondence with high-resolution CLXT and the emergence of the observed spatial mesoscale domain organization from a simple phenomenological framework presents interesting possibilities for further progress in understanding chromatin organization at sub-micrometer lengthscales. Interestingly, the connectivity between mesoscale domains—analyzed in both simulations and experiments—displays features characteristic of a marginally solid, jammed, polydisperse assembly^43,44^ with an average coordination number, $\langle z_c \rangle > z_{iso}$ ($z_{iso}=6$ is the isostatic condition for the coordination number distribution in 3D^44,45^). The alteration in the mesoscale domain size and the spatial arrangement of dense heterochromatin in perturbation experiments are also qualitatively captured by representing functional perturbations to heterochromatin 3D organization as disrupting the affinity that induces clustering. The precise mechanism of heterochromatin affinity is an open question and the subject of intense investigation. Numerous works have pointed to transient crosslinking between nucleosomes modified with lysine trimethylation, mediated by HP1$\alpha$ proteins and methyl transferase proteins, as the origin of heterochromatin droplet formation^2,9^. We represent the effect of these complex biochemical processes with a phenomenological net attraction between the HC/*B* beads, whose strength is determined by the relative stability of attached-detached states at the molecular level. Disruptions to the biochemical activity of these crosslinkers and mediators, or to the integrity of the chromatin fiber, are represented as a decrease in this net affinity, with the corresponding conditions displaying more diffuse chromatin, a trend consistent with that of the perturbation experiments (as seen Fig. S7).

Our direct visualization of functional heterochromatin states in intact hydrated states of cells opens up novel ways to correlate the cryo SXT molecular density maps with functional changes to the chromatin structure in diverse genome-wide studies. Functional characterization of spatially connected mesoscale domains can provide novel insights into mechanisms underlying transcriptional repression mediated by modular heterochromatin packing. Spatial heterogeneity in the molecular density of heterochromatin can also provide insights about its role in selective diffusion of regulatory factors into the heterochromatin domain. Collectively our experimental and theoretical analysis of the spatial organization of the heterochromatin structure, its internal arrangement into mesoscale domains, and the ability to map local and global functional chromatin state through CLXT provide an important mechanistic perspective on genome organization.

## Methods

### Experimental design

#### Experimental model

HMF3A and MCF7 cells were obtained from Applied Biological Materials Inc, Cat. No. T0153 and ATCC, Cat. No. ATCC HTB-22 respectively. HMF3A cells were stably transfected (Lipofectamine 3000) with fusion plasmid HP1$\alpha$ tagged with an enhanced green fluorescent protein (HP1$\alpha$-GFP) followed by 5 rounds of antibiotic selection (gentamicin G418) every 72 h to develop HMF3A$^{HP1\alpha -GFP}$ stable cell line. The MCF7 (ATCC, RRID: CVCL_0031), HMF3A$^{WT}$, and HMF3A$^{HP1\alpha -GFP}$ were grown in Dulbecco’s modified Eagle’s medium (DMEM) (Gibco) supplemented with 10% (volume [vol]/vol) FBS (Gibco) and 1% penicillin-streptomycin (Gibco) at 37 $^\circ$C in 5% CO$_2$ in a T25 cc flask. Cells used for these experiments were within the range of 10–15 passage numbers. For correlative imaging experiments, HMF3A cells were double transfected with (Lipofectamine 3000) with fusion plasmid for HP1$\alpha$ tagged with an enhanced green fluorescent protein (GFP) and fusion plasmid for *TRF*1 tagged with a red fluorescent protein (RFP). Carbon coated side of the grids, to culture cells, was treated with UV radiation for 3 h for sterilization and incubated with 1:50 (vol/vol) fibronectin solution for 30 min. Adherent cells were trypsinized, resuspended at a concentration of $1\times 10^6$ cells mL$^{-1}$, and seeded onto fibronectin-coated grids and left undisturbed overnight.

#### Pharmacological treatments

To inhibit the activity of HDAC, we treated grid-plated cells with 5 $\upmu$M of TSA (Sigma-Aldrich) in DMSO(Sigma-Aldrich), directly mixed in the cell culture media, for the duration of 24 h, prior to cryo fixation. To inhibit the activity of G9a Histone Methyl Transferase (HMT) (Sigma-Aldrich), cells were treated with 5 $\upmu$M of BRD4770(Sigma-Aldrich) in DMSO directly mixed in cell culture media for 24 h. For reactive oxygen species stimulation, cells were treated with Phenyl 2-pyridyl ketoxime (PPKO) (Sigma-Aldrich) at the concentration of 1 *m*M in DMSO for 24 h.

#### Room temperature fluorescent imaging for CLXT

To correlate the light microscopy images taken at room temperature with cryogenic soft X-ray tomography, single cells on the gold Quantifoils were captured with EVOS M5000 wide-field microscope with a Z-step size of 1 $\upmu$m using a 40$\times$, 0.65 NA objective prior to plunge freezing. 3D images of regions were deconvolved using experimentally obtained point spread function on 200 nm fluorescent beads. The high degree of correspondence with correlation accuracy of 800 nm was observed using this methodology (Fig. S1D,E).

#### CLXT sample preparation

To track the cells between light and soft X-Ray modalities, we used gold Quantifoil 2/2 holey carbon-film microscopy finder grids for correlative imaging of cells. Prior to cryo fixation, all the samples from the control and treatment conditions were washed with DPBS for 30 s to remove the excess media and enable maximum X-ray transmission. 1.5 $\upmu$L of fiducial markers (100 nm of gold nanoparticles; BBI Solutions, UK) were added on top of the grid for tilt series alignment to a common axis of rotation. Grids were then blotted for 0.5 s to 2 s depending on the cell density and rapidly vitrified using plunge freezing (Leica EMCPC) and stored in liquid nitrogen thereon.

#### Cryo-CLXT imaging

Grids were screened with a Linkam CMS196 cryo-stage on a Zeiss Axioscope to choose the best grids in terms of cell density and ice thickness using a 10$\times$ objective for CLXT imaging. Based on the 10$\times$ images, regions of interest containing 1–3 cells per grid square mesh were selected to avoid the superposition of cells during tilt series acquisition. These selected Regions of Interest (ROI) were imaged using 50$\times$ objective at 405 nm, 428 nm, and 555 nm wavelengths of visible light. Samples were transferred to MISTRAL Beamline^46^ at ALBA under cryogenic conditions. We acquired a Soft X-ray tilt series by irradiating the sample with X-Rays of 520 eV of energy before the oxygen K edge absorption, using a 40 nm Zone Plate. Based on the selected sub-nuclear region for the field of view, pixel size was set at 13 nm. Tilt series were acquired over a limited angle range of − 65 to 65 degrees with a regular angular step of 1$^\circ$ with a missing wedge. Limited angle Cryo-SXT leads to the elongation of features in the z-axis. Projections were normalized with the incoming incident photon flux and deconvolved with the measured point spread function of the Transmission X-ray Microscope. The total radiation dose during the tilt series acquisition was well below the maximum recommended dose to avoid radiation damage^22^.

#### Tilt series alignment and linear absorption coefficient (LAC) reconstruction

Tilt series projections were aligned using gold fiducial particles by building a seed model on IMOD^47^ and successively reducing the alignment error by visual inspection. The transmission intensity of soft X-rays of intensity *I* and impinging intensity $I_0$ passing though media of thickness $t_n$, is related to the mass absorption coefficient $\mu _n(x,y,z)$ and volumetric density $\rho _n(x,y,z)$ as

1 $$\begin{aligned} I=I_0 e^{\int \mu _n\rho _ndt_n} \end{aligned}$$

Assuming nuclear regions as perfectly absorbing medium, local *LAC*(*x*, *y*, *z*) is a spatially dependent empirical quantity referred to as the product of mass absorption coefficient $\mu _n(x,y,z)$ and volumetric density $\rho _n(x,y,z)$ and is given by:

2 $$\begin{aligned} \int LAC(x,y,z)dt_n=-\ln \frac{I}{I_0} \end{aligned}$$

For the TXM images with *i* projections to reconstruct the discretized volume of digital tomogram with the total reconstructed voxels, $N_{vox}$, Eq. (2) can be expressed as^48^:

3 $$\begin{aligned} \sum _{j=1}^{N_{vox}} w_{i,j}LAC(j)=-\ln \frac{I_i}{I_0} \end{aligned}$$

Here, $w_{ij}$ $\in [0,1]$ is the weighting function. The algebraic solution of the *LAC*(*j*) tomogram given by Eq. 3 was obtained iteratively on the aligned tilt series and corresponding corrected angular intervals using SIRT reconstruction provided by TOMO3D^49^ with 20 iterations. Prior to reconstruction, we impainted the fiducial markers from the aligned tilt-series using the impainting method outlined in previous studies^50^ to avoid streaking artifacts. All the reconstructed tomograms used for this study are presented in Fig. S3A. Tomograms presented in the figures are displayed with a greyscale colormap with higher LAC corresponding to darker color shades.

#### Fourier shell correlation (FSC) resolution

We characterized the resolution of the 3D reconstruction of the segmented nuclear volumes by using the even-odd Fourier shell correlation method^51^ by dividing our tilt series individual stack of 65 images followed by SIRT reconstruction, generating even tomogram, *E*(*x*, *y*, *z*) and odd tomogram *O*(*x*, *y*, *z*).We calculate the Fourier transform, *F*(*k*), *G*(*k*) respectively, and perform the spatial correlation using the relation on the reconstructed raw volume.

4 $$\begin{aligned} \textbf{FSC}_{FG}^{(i)}(k) =\frac{\sum \limits _{l,m,n \in R(k)}\Re \{F^{(i)}_{l,m,n}G^{*(i)}_{l,m,n}\}}{\left( \sum \limits _{l,m,n \in R(k)} \mid F^{(i)}_{l,m,n} \mid ^2\sum \limits _{l,m,n \in R(k)} \mid G^{(i)}_{l,m,n} \mid ^2 \right) ^{1/2}} \end{aligned}$$

The maps generated through half-tilt series reconstruction are generally noisier than the full-series reconstruction.Therefore, we estimate the approximate experimental resolution for our data as previously described^52^.

5 $$\begin{aligned} \textbf{FSC}'_{e/o}(k)=\frac{2\textbf{FSC}(k)}{\textbf{FSC}(k)+1} \end{aligned}$$

### Image analysis

#### Segmentation and LAC quantification

The intensity values obtained after the iterative reconstruction of the tilt series images are the voxel-wise linear absorption coefficient (LAC) of the sample which is indicative of the molecular composition of the sample. For our imaging conditions, at the irradiation energy of 520 eV and the voxel edge length of 13 nm for the CLXT imaging, the LAC values correspond to the concentration of carbon in every voxel volume of 2197 nm$^3$^53^. By visual inspection, nuclear boundaries, nucleoplasmic ultrastructure, and euchromatin can be identified based on their shape, texture, or LAC values. Via our CLXT imaging workflow, we validate that the dense regions inside the HMF3A nucleus correspond to Heterochromatin with LAC values ranging from 0.1-0.25 $\upmu$m$^{-1}$. Perinucleolar heterochromatin in HMF3A cells forms distinct boundaries and can be identified in the control, TSA-treated and G9a-inhibited nuclei visually, with ROS-stimulated sample we find the outer periphery of the heterochromatin regions are not well defined. For the segmentation task of the nucleolus, euchromatin, nuclear boundary, and heterochromatin nodes for all the tomograms, we traced the boundaries of each region using hand annotations using the *volumeSegmenter* tool from MATLAB and refined our annotations by 2D active contour function *activecontour* from MATLAB for each annotation in the orthonormal plane. We interpolated between each hand-segmented label using the Delaunay triangulation *delaunayTriangulation* function from MATLAB. We visualized the LAC values of the segmented volume using the plotly3d^54^ library for MATLAB for volume rendering by keeping the piece-wise linear transparency profile ramping in opacity from lower to higher LAC values as shown in Fig. 2C. The depth of field of the zone plate (Outer ring diameter = 40 nm) is 3.3 $\upmu$m^12^, conservatively providing us with an optimal z-range of 1.2 $\upmu$m with all the reference tilt series regions in focus. For our analysis, we ignored the segmented nuclear regions that lie outside this range.

#### Heterochromatin mesoscale domain segmentation

We segmented the connected clusters of heterochromatin mesoscale domains by thresholding the molecular density maps obtained from LAC value characterization of heterochromatin regions using the following relation

6 $$\begin{aligned} \pmb {\Theta }_{HMD}=\min (LAC)+0.6.(\max (LAC)-\min (LAC)) \end{aligned}$$

Here $\pmb {\Theta }_{HMD}$ is the selected threshold, $\max (LAC)$ and $\min (LAC)$ are the maxima and minima of the LAC values of the segmented heterochromatin regions respectively. We mapped the molecular density maps back to the thresholded regions and applied the watershed algorithm to obtain the domain boundaries based on the distance transform. To account for the missing wedge effect, we rescale the dimension of segmented mesoscale domains in the z-axis using a factor $f_{mw}=D_{rec}/D_{fid}$, where $D_{rec}$, $D_{fid}$ are the diameter of fiducials measured in the reconstructed tomograms and their reported dimension. The workflow of this approach is presented in Fig. S2B and the characterized heterochromatin mesoscale domain size distribution given by effective diameter, $D_{eff}= \left( \frac{6f_{mw} V_{seg}}{\pi }\right) ^{1/3}$ presented in Fig. 2D, where $V_{seg}$ is the volume of the segmented heterochromatin mesoscale domain.

#### Light and X-ray image registration

Light and Cryo-SXT data were aligned using the segmented mask of the nucleus obtained from each of the modalities labeling the region of interest. We obtain the boundaries of the mask using the *bwboundaries* function from MATLAB for both the light microscopy and Cryo-SXT data. We then find the optimal isotropic affine transformation using the *imregconfig* function from MATLAB to allow for isotropic rescaling, rotation, and translation correction of light microscopy data. We have quantified the efficacy of our method (Fig. S1C) for both cryo-temperature and room-temperature (37 $^\circ C$) light microscopy correlated with cryogenic TXM images.

### Polymer model

#### Simulations

The bidisperse polymer model is defined as consisting of HC/*B* beads (representing groups of nucleosomes with H3K9me3/H3K27me3 marks) and EC/*A* beads (that do not contain such heterochromatin-associated marks). HC/*B* beads size is set at 25 nm using the scaling argument detailed below. The smallest mesoscale domain sizes for dense heterochromatin observed in Figs. 1 and 2 are of $\approx 50$ nm. Assuming that the smallest segmented mesoscale domains correspond to the smallest identified cluster in the simulations of 4 HC/*B* beads equidistant from each other, we consider the HC/*B* beads to be half this size, 25 nm (assuming a tetrahedral arrangement where 4 beads are equidistant) and investigate the resultant clustering into mesoscale HC/*B*-rich domains. Considering the size of a nucleosome to be 10 nm and the number of nucleosomes, $N_{nucleo}$, to be related to the size of the bead, $d_{bead}$, and the size of the nucleosome, $d_{nucleosome}$, as $N_{nucleo}\propto (d_{bead}/d_{nucleosome})^{3}$, each HC/*B* bead corresponds to 16 nucleosomes or approximately 3 kbp. While HP1$\alpha$ and methyl transferases are understood to work in a positive feedback mechanism to produce contiguous regions of heterochromatin, contiguous unmethylated/acetylated regions are typically smaller, as evidenced also in super-resolution studies^11^. We consider EC/*A*-type beads to have a diameter of 18 nm, corresponding to $\approx 6$ nucleosomes or 1.2 kbp of DNA. Given the segment lengths corresponding to our bead pairs exceed the reported persistence length of chromatin ($\approx 30$ nm^55^), we neglect any bending rigidity-related energy cost. Visualizations from simulation trajectories are performed using OVITO^56^.

#### Simulation conditions

We choose a ratio of 55:45::*A*:*B* beads in a spherical cavity of constant volume, considering number densities, $N/V\approx 0.2$, within the meaningful range of 6.2 Gbp of DNA in a nuclear cavity of diameter 1–10 $\mu$m. The dense nodes observed in CLXT have typical diameters of 0.5–1 $\upmu$m and we we consider polymer chains of $N=3000$ beads in a cavity of diameter $\approx 1~\mu$m. We use physiologically relevant values of temperature, energy, and viscosity, described in Ref.^19^ to specify a set of reduced units. $T = 310$ K, and we consider energy unit as $e = 1 k_B T = 4.28$ pN$\cdot$nm. The frictional drag on monomers is approximated by Stokes’ law, and the corresponding viscosity (of the nucleoplasm) is assumed to be $\eta =1.5$ cP. Considering the nucleoplasmic viscosity as unity in reduced units, i.e.m $\eta ^{*}=1$, we get a reduced time unit of $\tau = \frac{\eta \sigma _{AA}^3}{\eta ^{*} e} = 0.1435\times 10^{-3}$ ms.

#### Interactions

We use the set of interactions introduced in Ref.^19^. The set of interactions experienced by each bead thus consists of (i) soft Gaussian “blob” exclusion $h_{vex}$^35^ without any hard cores (ii) a FENE attraction between neighboring beads along the polymer chain, (iii) attraction between all HC/*B* beads (iv) repulsion from the walls of the spherical confining cavity of the Weeks–Chandler–Andersen type^57^. An interaction cut-off is specified as $3\sigma _{\alpha \beta }$ for the exclusion and heterochromatin attraction potentials.

7 $$\begin{aligned} h_{vex}^{ij} = {\left\{ \begin{array}{ll} \epsilon _{vex} \exp (-\alpha _{vex} r_{ij}^2) & \text {if }r_{ij} \le r_{cut}\\ 0 & \text {otherwise} \end{array}\right. } \end{aligned}$$

for particles *i* and *j* within the interaction cut-off, $r_{cut}$. The finite extensible non-linear elastic interaction

8 $$\begin{aligned} h_{FENE}^{ij} = {\left\{ \begin{array}{ll} -12 kr^2_0 \ln \left[ 1 - \left( \frac{r_{ij}}{r_0}\right) ^2\right] & \text {if }|j-i| = 1\\ 0 & \text {otherwise} \end{array}\right. } \end{aligned}$$

The heterochromatin affinity is modeled using an attractive interaction adjusted to maintain the optimal separation between interacting HC/*B* beads.

9 $$\begin{aligned} h_{HC}^{ij} = {\left\{ \begin{array}{ll} -\epsilon _{HC} r_{ij}^2 \exp \left( -\alpha _{HC} \left[ d_B - \frac{1}{\alpha _{HC} d_B} - r_{ij}^2\right] \right) & \text {if } r_{ij} \le r_{cut}\\ 0 & \text {otherwise} \end{array}\right. } \end{aligned}$$

$$\begin{aligned} h_{wall}^{i} = {\left\{ \begin{array}{ll} -4\epsilon _{wall}\left[ (\frac{\sigma _i}{r_iw})^{12} - (\frac{\sigma _i}{r_iw})^6 \right] & \text {if }r_{iw} \le 2^{1/6}\sigma _i\\ 0 & \text {otherwise} \end{array}\right. } \end{aligned}$$

The sum of the interactions over all particles and pairs gives the Hamiltonian

10 $$\begin{aligned} H_i = \sum _j \left[ h_{vex}^{ij} + h_{FENE}^{ij} + h_{HC}^{ij} \right] + h_{wall}^{i} \end{aligned}$$

The values of the parameters used in the simulations are included in the Supplementary Materials in both reduced and real units.

### Initialization and equations of motion

The system is initialized by placing the beads as a self-avoiding random walk within the spherical cavity. Given that the interaction is a soft repulsion, large overlaps are not prevented. The overdamped Langevin equation

11 $$\begin{aligned} \frac{d\mathbf {x_i}}{dt} = -\frac{1}{6\pi \eta (\sigma _i/2)}\frac{\partial H_i}{\partial \mathbf {x_i}} + \sqrt{\frac{2k_BT}{6\pi \eta (\sigma _i/2)}} \zeta _\textbf{t} \end{aligned}$$

is integrated using the Euler–Maruyama scheme using a timestep of $10^{-4}\tau$. Convergence is determined by monitoring the energy as a function of time and identifying the time at which a steady state is achieved. The mean squared displacement and overlap functions are computed (see Supplementary Materials) using configurations in steady state as initial points. Snapshot configurations are stored in well-spaced time intervals to identify clusters for HC/*B* beads and compute other structural properties.

#### Mean squared displacement and overlap function

The mean squared displacement is next computed as

12 $$\begin{aligned} \langle r^2 \rangle _{\alpha } = \frac{1}{N_{\alpha }}\sum \limits _{i=1}^{N_{\alpha }} \left( \mathbf {r_i^2}(\textbf{t}) - \mathbf {r_i^2}(\textbf{0}) \right) , \end{aligned}$$

where $\alpha ~\in [A,B]$ denotes the bead type.

The overlap function, *Q*(*t*), is defined as the fraction of beads that have not moved more than one bead diameter (bead diameter is bead-type specific) relative to an initial position, at a given time interval. This can be written as:

13 $$\begin{aligned} Q(t) = \frac{1}{N_{\alpha }}\sum \limits _{i=1}^{N_{\alpha }} \theta \left( \sigma _{\alpha } - |\mathbf {r_i}(\textbf{t}) - \mathbf {r_i}(\textbf{0})|\right) \end{aligned}$$

where $\alpha ~\in [A,B]$ again denotes the bead type and the step function, $\theta (x) =1$ if $x>0$ and $\theta (x)=0$ if $x\le ~0$.

#### Radial distribution function

The radial distribution function is computed in confinement by first computing the histogram of pairwise distances for either EC-EC/$A-A$, EC-HC/$A-B$, or HC-HC/$B-B$ pairs of beads. The histogram of pairwise distances is then normalized against the background density of the unstructured “ideal gas” at the same number density as a result of which the radial distribution function has unit value at large *r*. Care must be taken in confinement conditions when computing the background normalization factor given the absence of any periodic images. In spherical confinement, we identify the volume of the shell of thickness $[r,r+\delta r]$ that intersects with the confinement volume, $V_{shell}$, in order to normalize the histogram count with the ideal gas normalization factor, $\rho \times V_{shell}$.

#### Cluster definition and connectivity in polymer simulations

Pairs of HC/*B* beads within a cut-off separation—the first minimum of the bead radial distribution function (see Supplementary Materials)—of each other are defined as being “bonded”. Considering a HC/*B* bead surrounded by at least 3 other HC/*B* beads, we then construct the contiguous clusters of such HC/*B* beads. Spatially separated clusters that are directly linked by segments of the polymer chain are considered to be neighboring. We thus define the coordination number of a cluster as the number of other such clusters to which the given cluster is connected. Note that multiple connecting segments are not multiply counted in our definition. Additionally, we treat clusters within $\sigma _{BB}$ of the wall as having one additional neighbor contact/constraint. The total number of such connections is defined as the coordination number, $z_c$.

#### Coordination number characterization in SXT

For the noncrystalline structure the coordination number $z_c$ is defined as the area under the first peak of the radial distribution, ranging from $r_1$, the radius of the reference particle, to $r_2$, the edge of the first peak of the radial distribution function *g*(*r*) and it is given by the relation^58^:

14 $$\begin{aligned} z= 4\pi \rho _{MD}\int _{r1}^{r2} r^2 g(r)dr \end{aligned}$$

Here $\rho _{MD}$ is the bulk number density of mesoscale domains. The value, $r_2$ was numerically obtained from the *g*(*r*) function computed for the experimentally obtained connected clusters of heterochromatin architecture. The nearest neighbor distance distribution is the pairwise distance calculated between the centroids of all the segmented mesoscale domains.

### Statistical analysis

All statistical analyses and plotting were carried out in R^59^. For box plots, the box limit represents the 25th to 75th percentile and whiskers $1.5\times$ interquartile range. We evaluated the statistical significance of the mean with the Mann-Whitney U-test, performed between a sample of interest and the corresponding control. *P<0.05; **P<0.01; ***P<0.001; ****P<0.0001. To establish the consistency in results obtained across multiple beamtimes and batches we proposed a multiparametric analysis using descriptive morphological features. We establish the baseline for accuracy in inter-class separability using LDA and validate it with non-linear classification using Random Forest from *MASS* library, feature reduction was done by PCA using the *prcomp* library. The scores from the first twelve principal components were then used as discriminant variables in LDA. We used the LDA classifier trained on 60% of the data to separate the different classes of treatment conditions. A Random Forest classifier was first tuned with the optimal numbers of decision trees and trained on $60\%$ of the data. The descriptive features with accuracy scores greater than $40\%$ of the maximum value were selected. The $95\%$ confidence ellipsoids in the LDA plots were evaluated using the *ggplot* library. Statistical data visualization and representation are carried out using *ggplot* library.

## Supplementary Information

Supplementary Information 1.

Supplementary Information 2.

Supplementary Information 3.

Supplementary Information 4.

Supplementary Information 5.

Supplementary Information 6.

Supplementary Information 7.

Supplementary Information 8.

Supplementary Information 9.

Supplementary Information 10.

Supplementary Information 11.

Supplementary Information 12.

Supplementary Information 13.

Supplementary Information 14.

## Data Availability

All data are available in the main text or the supplementary materials. Further data are available from the corresponding author upon reasonable request.
