# Supplementary material for: Correlative light and soft X-ray tomography of in situ mesoscale heterochromatin structure in intact cells
Source: Sci Rep. 2024 Nov 12;14:27706. doi: 10.1038/s41598-024-77361-2 (PMC11557596; doi:10.1038/s41598-024-77361-2)
Supplement: Supplementary file 1 — Supplementary Information 1. [file 41598_2024_77361_MOESM1_ESM.pdf]

Supplementary Material for

Correlative light and soft X-ray tomography of *in situ* mesoscale heterochromatin structure in intact cells

Rajshikhar Gupta,[1,2] Yagyik Goswami,[1] Luezhen Yuan,[1,2] Bibhas Roy,[1]
Eva Pereiro,[3] GV Shivashankar[1,2*]
[1]Laboratory of Nanoscale Biology, Paul Scherrer Institut, Villigen, Aargau, Switzerland
[2]Department of Health Sciences and Technology, ETH Zürich, Zürich, Switzerland,
[3]ALBA Synchrotron Light Source, Cerdanyola del Vallés, Barcelona, Spain
[*]To whom correspondence should be addressed; E-mail: gshivasha@ethz.ch

Simulation parameters:

Table I below has the parameters in reduced and real units that were used in the simulations.

Parameter	Reduced units	real units
σ_{AA}	1	16 nm
σ_{BB}	1.32	25 nm
σ_{AB}	1.16	18.56 nm
ϵ^{vex}_{AA}	6.6539	28.48 pN $\cdot$ nm
ϵ^{vex}_{BB}	11.507	49.25 pN $\cdot$ nm
ϵ^{vex}_{AB}	8.9153	38.16 pN $\cdot$ nm
α_{AA}	1.47153	0.0058 nm^{-2}
α_{BB}	0.8466	0.0033 nm^{-2}
α_{AB}	1.0927	0.0043 nm^{-2}
α_{HC}	3.825	0.00149 nm^{-2}
$k_{stretch}$	3.3833	14.48 pN$\cdot$ nm
η	1	1.5 cP
T	1	310 K
τ	1	0.1435×10^{-3} ms

TABLE I: List of parameters in the interaction potential and their values in real and reduced units

Structural characterization

Descriptive feature list

We present the list of features for the multiparametric analysis of the mesoscale organization of heterochromatin across treatment conditions. We have divided these features into three categories to describe the 3D spatial LAC distribution, mesoscale domain organization, and 2D/3D morphology of the entire heterochromatin region and the individual mesoscale domain, schematically represented in Fig. S4 A, B.

- Descriptive features: heterochromatin region
  - 2D Morphometrics: Projection of segmented heterochromatin volume
    1. Aspect ratio, area, bounding box height, bounding box width, maximum and minimum caliper distance, roundness, shape factor, circularity, concavity of 2D Projection of heterochromatin volume.

2. Mean and standard deviation of centroid to edge distance of 2D projection of heterochromatin volume.
3. Semi-major axis length of the fitted ellipse, Semi-minor axis length of the fitted ellipse to the 2D projection of heterochromatin volume.

– 3D Morphometrics: Heterochromatin volume

1. $R1$, $R2$, $R3$ semi-axes length of ellipsoid fit to 3D heterochromatin volume.
2. Volume, compactness, elongation, ferret distance and Flatness of 3D heterochromatin volume.
3. Maximum, mean, minimum, and standard deviation of 3D heterochromatin surface to centroid distance.
4. Sparseness, sphericity, surface area of heterochromatin volume.
5. Volume fraction of heterochromatin volume to bounding cuboid and ellipsoid.
6. Volume of bounding ellipsoid to heterochromatin volume.

– Spatial LAC distribution

1. Mean, median, mode and standard deviation of range normalized LAC.
2. Variance, Skewness, Entropy, Kurtosis, Standard deviation, Volume normalization of values of LAC.
3. Distance between geometric vs LAC weighted centroid, Ratio of number voxels with Highest LAC to Lowest LAC, Ratio of a number of voxels with LAC value greater than 80 percentile to less than 20 percentile.

– Mesoscale domain organization

1. Number of mesoscale domains, Ratio of Mesoscale domain volume, and total heterochromatin volume.
2. Maximum, mean, median, minimum, and standard deviation of
   (a) $D1$: inter mesoscale domain centroid to centroid distance,
   (b) $D2$: inter mesoscale domain surface to surface distance.
3. Maximum, mean, median, minimum, and standard deviation, and a total of
   (a) $D3$: mesoscale domain centroid to heterochromatin centroid distance,
   (b) $D4$: the nearest distance of mesoscale domain surface to heterochromatin centroid,

(c) $D5$: the nearest distance of mesoscale domain surface to heterochromatin surface,

(d) $D6$: the radial distance between mesoscale domain surface to heterochromatin surface,

(e) $F1$: the ratio of $D3$ to radial distance R,

(f) $F2$: the ratio of $D6$ to radial distance R,

(g) Mesoscale domain volume.

- Descriptive features: mesoscale domains
  - Principal axis length 1,2, and 3, surface area, volume equivalent diameter, the extent of mesoscale domain
  - Mean, minimum, maximum of LAC and distance between the geometric center and LAC weighted center of the mesoscale domain
  - Mean, minimum, maximum, standard deviation, and a total of inter-particle pairwise distance, IPD for the mesoscale domain with other surrounding domains.

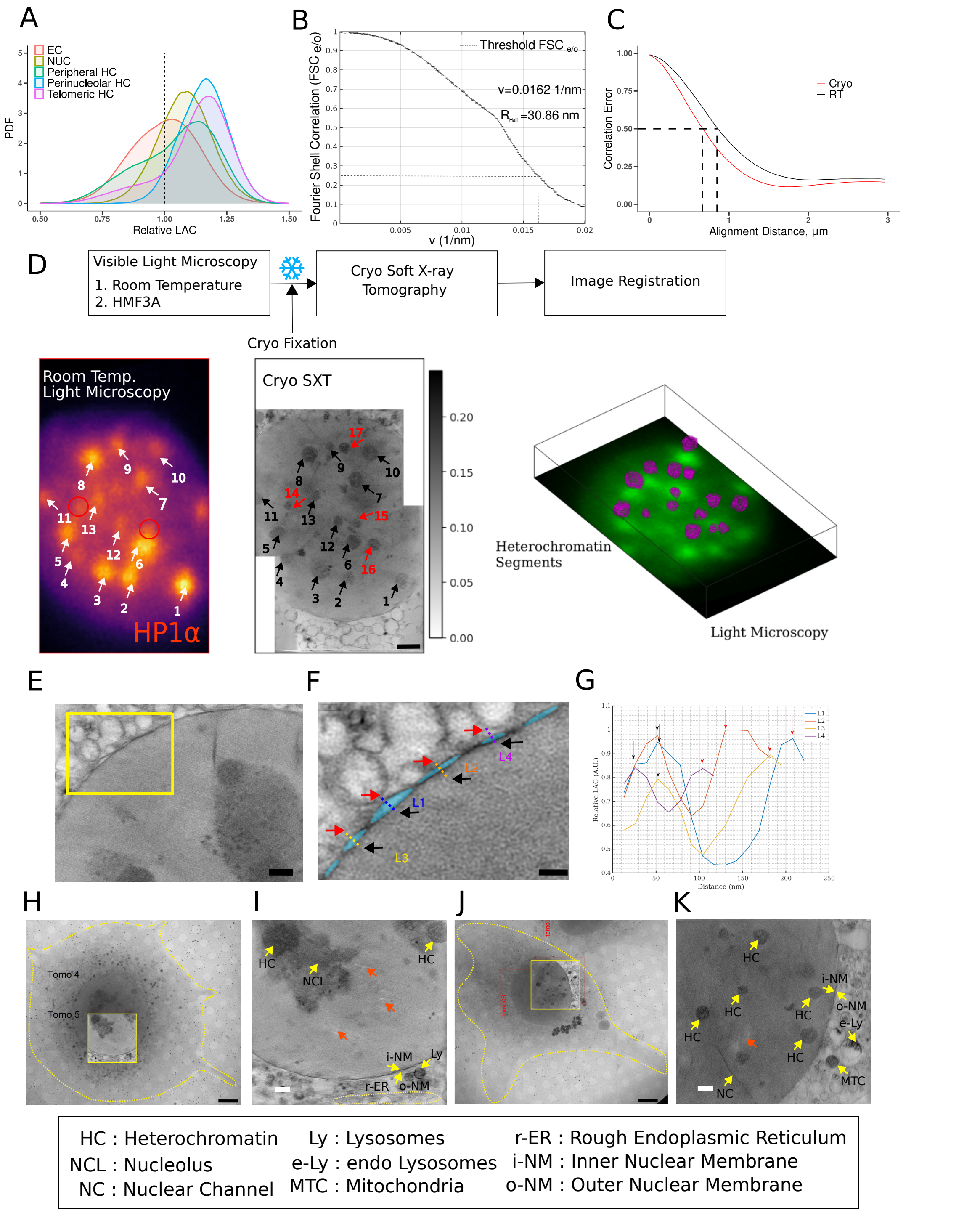

FIG. S1: Characterization of CLXT. (**A**) LAC values of regions segmented from cryo-SXT, EC: Euchromatin, NUC: Nucleolus, Peripheral, Perinucleolar, and Telomeric Heterochromatin normalized by the average LAC of the nucleus. (**B**) The FSC curve represents the half-pitch resolution of 30.86 nm for cryo-SXT tomography, the error bar represents standard error for a particular spatial frequency. (**C**) Correlation Error between Cryo-SXT with light microscopy at room temperature (37 °C) and Cryogenic condition. (**D**) Workflow and representative image for room temperature light microscopy of HP1α -GFP labeled HMF3A cell, Max-Z-projection of SXT, and 3D representation of heterochromatin regions. (**E**) LAC virtual slice from one of the cryo-SXT FOV. The yellow annotation marks the region at the nuclear periphery. (**F**) The zoomed region from the LAC virtual slice clearly shows the dual membranes of the nucleus. The colored annotation is a cut section perpendicular to the membrane, The red and black annotations mark the outer and inner membrane respectively. The blue annotation shows a contiguous dual membrane separated by nuclear pores. (**G**) The relative LAC values are plotted along the yellow cut section showing clear peaks corresponding to the inner and outer membranes for different line cuts. (**H**),(**J**) The TXM projection for the whole HMF3A^{WT} cell showing, overlayed with the virtual slice from corresponding cryo-SXT. The dotted yellow annotation marks the cell boundary. Scale bar: 5 μm. (**I**),(**K**) Zoomed-out virtual slice showing characteristic mesoscale heterochromatin organization in HMF3A^{WT} cells. Red arrows label the central spot artifact. Scale bar: 1 μm Scale Bars: (**D**) 2μm, (**E**) 1μm, (**F**) 0.5 μm, (**H,J**) 5 μm, (**I,K**) 1 μm

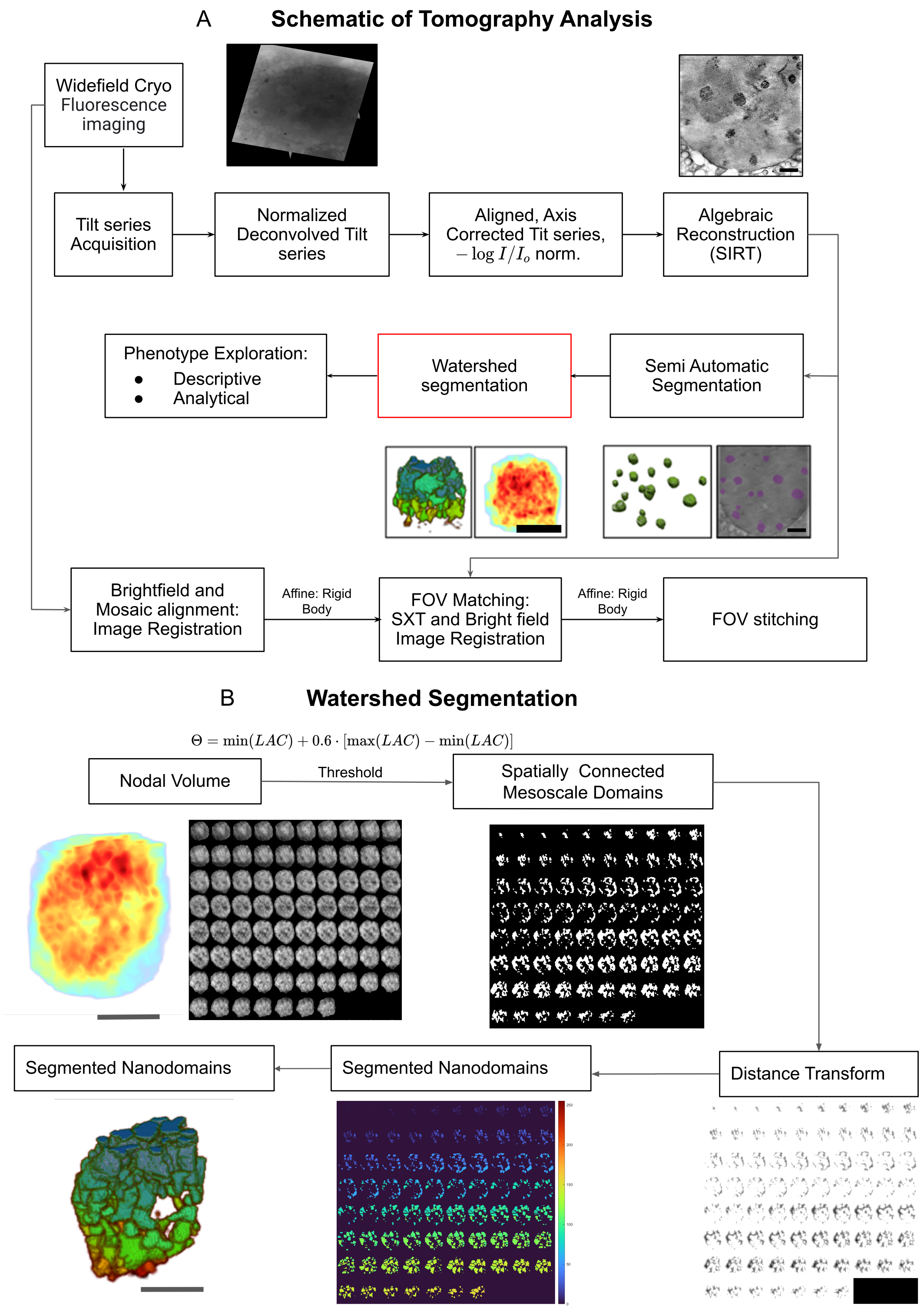

FIG. S2: (**A**) Detailed schematic of CLXT framework for visualization of heterochromatin structure *in situ*. (**B**) Step-by-Step visual representation of segmentation of dense mesoscale domains in the cryo-SXT data. Scale Bars: (**A**) SIRT SXT FOV 2μm, heterochromatin domain 1μm (**B**) 1μm.

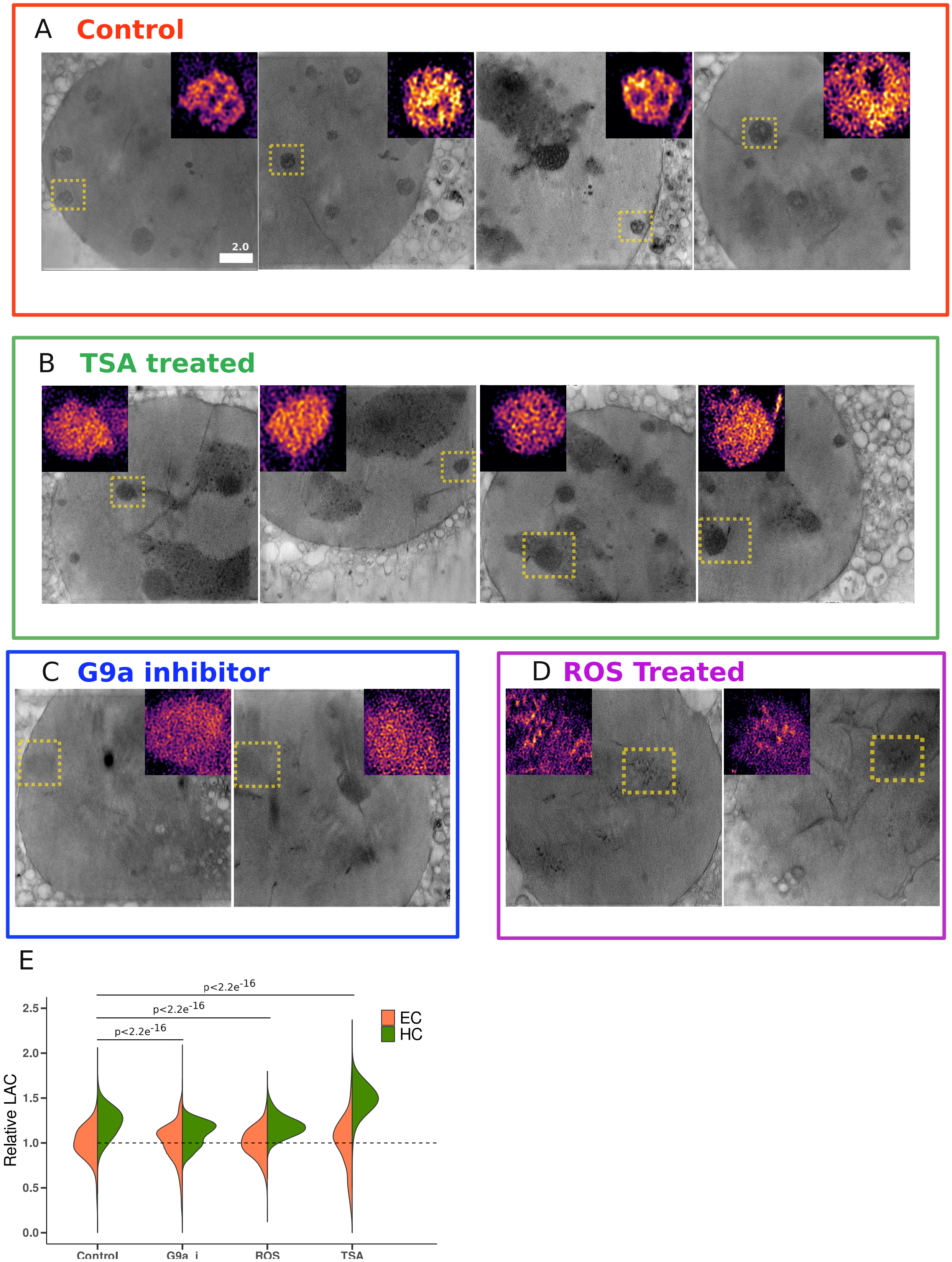

FIG. S3: Representative virtual slices of LAC tomograms from treatment conditions. (**A**) Control (**B**) TSA treated (**C**) G9a-i (**D**) ROS stimulated, Inset: zoomed in- local range color mapped regions of interest marked as yellow rectangle indicating heterochromatin regions in HMF3A^{WT} cells. (**E**) LAC of heterochromatin and Euchromatin regions normalized by the average LAC of the nucleus for all the represented measurements. Scale Bar: 2 μm.

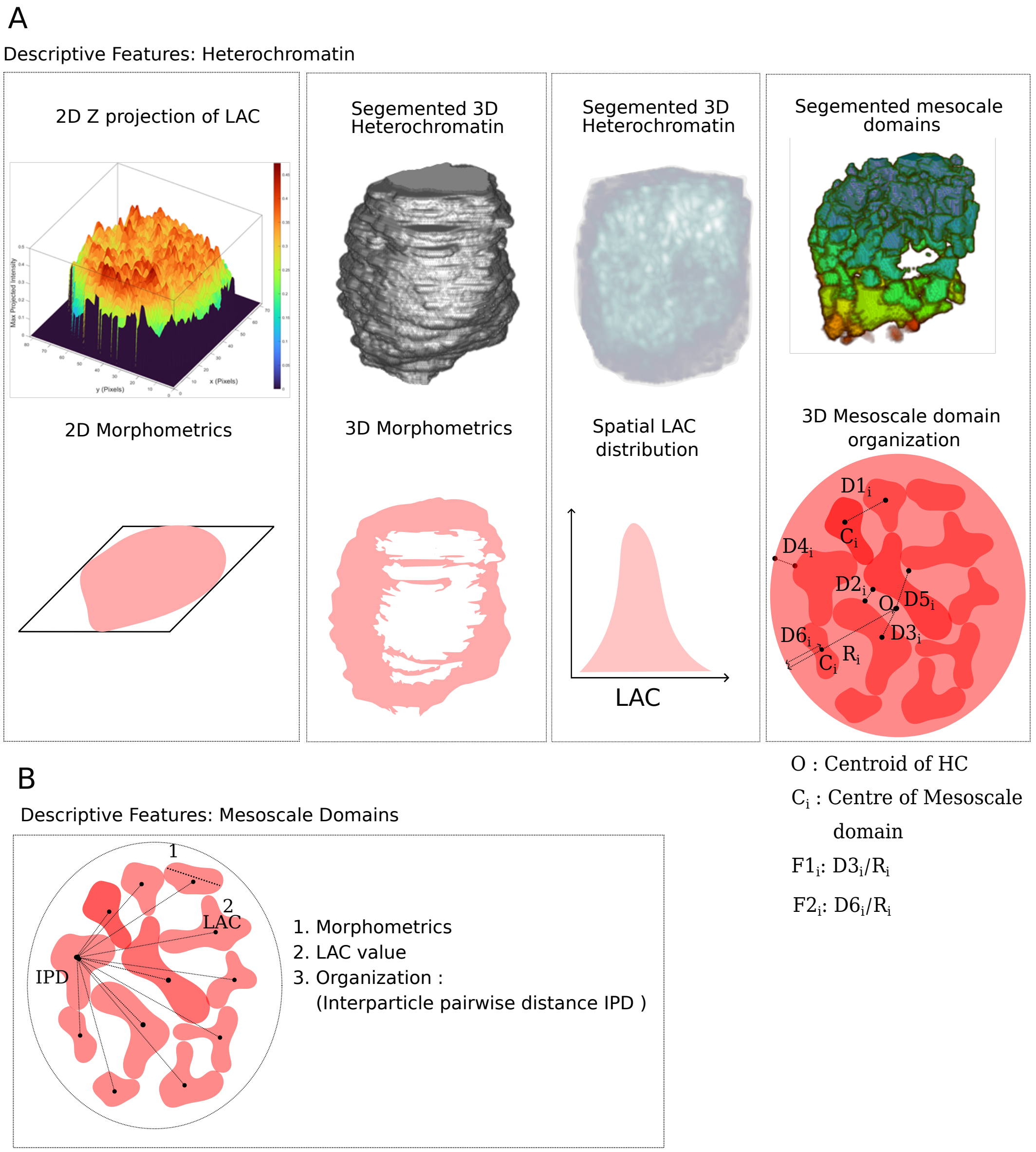

FIG. S4: Descriptive feature analysis of heterochromatin regions and mesoscale domains. (**A**) Schematic of different categories of descriptive features to analyze class differences in heterochromatin organization across treatment conditions. (**B**) Schematic of features used to analyze changes in mesoscale domains in all the treatment conditions, against control.

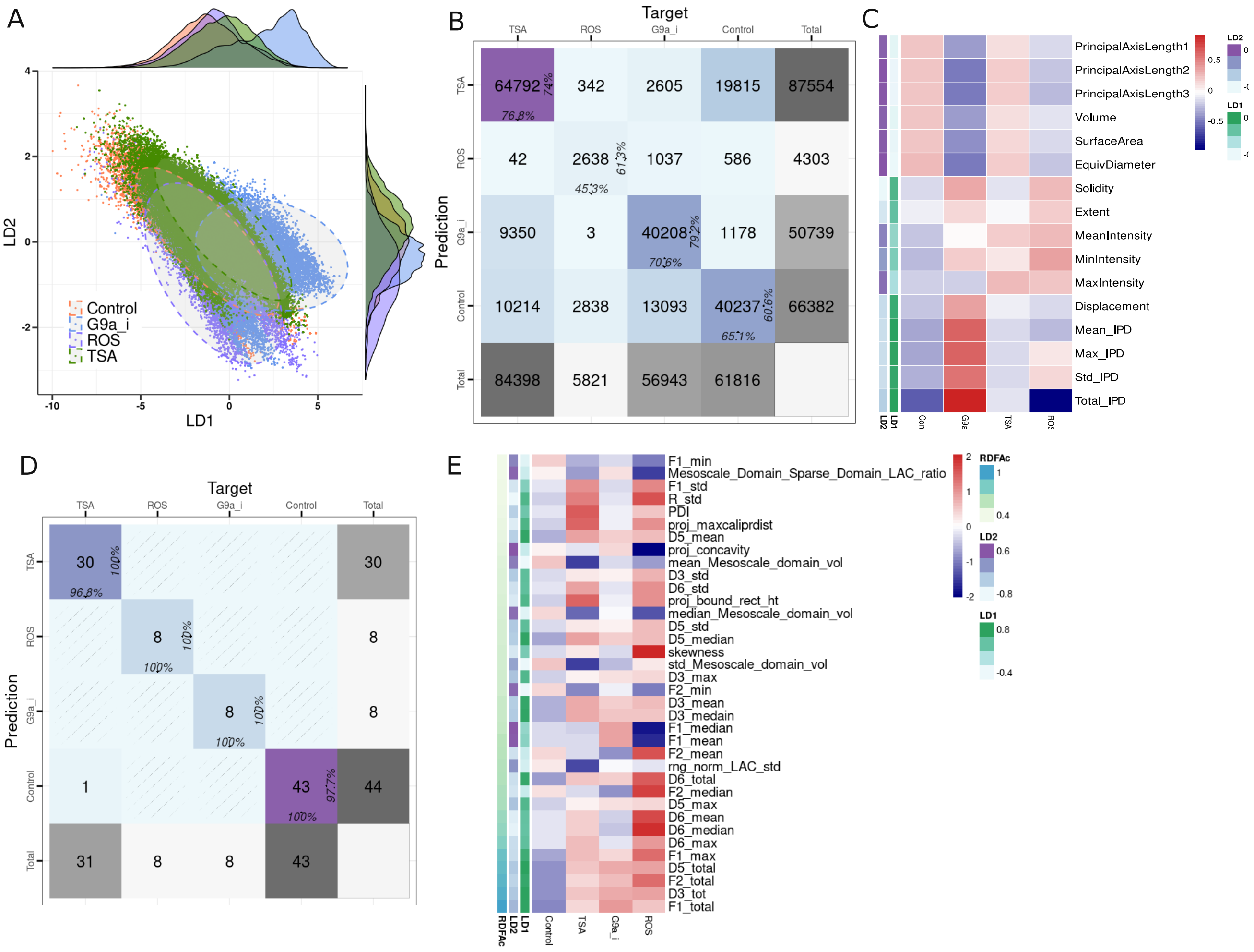

FIG. S5: (**A**) Linear Discriminant analysis (LDA) of multi-parametric descriptive features for mesoscale domains showing subtle inter-class separation across all the treatments, indicated by a 95% confidence level for a multivariate t-distribution. (**B**) Confusion matrix characterizing the total number of true and false classifications made by LD classifier trained on the descriptive features of the mesoscale domain in each treatment condition on the testing dataset. (**C**) Heat map of descriptive features of mesoscale domains sorted according to its correlation with LD1. (**D**) Confusion matrix characterizing the total number of true and false classifications made by LD classifier on the testing dataset, trained on the descriptive features of the Heterochromatin domain in each treatment condition. (**E**) Heatmap of selected descriptive features of Heterochromatin domain sorted according to the Random Forest (RF) accuracy score. Color Guides- LD1: Linear Discriminant 1, correlation coefficients, LD2: Linear Discriminant 2 correlation coefficients, RF-Ac: Random Forest accuracy score, Heat Map Intensity: Value of features scaled by the range.

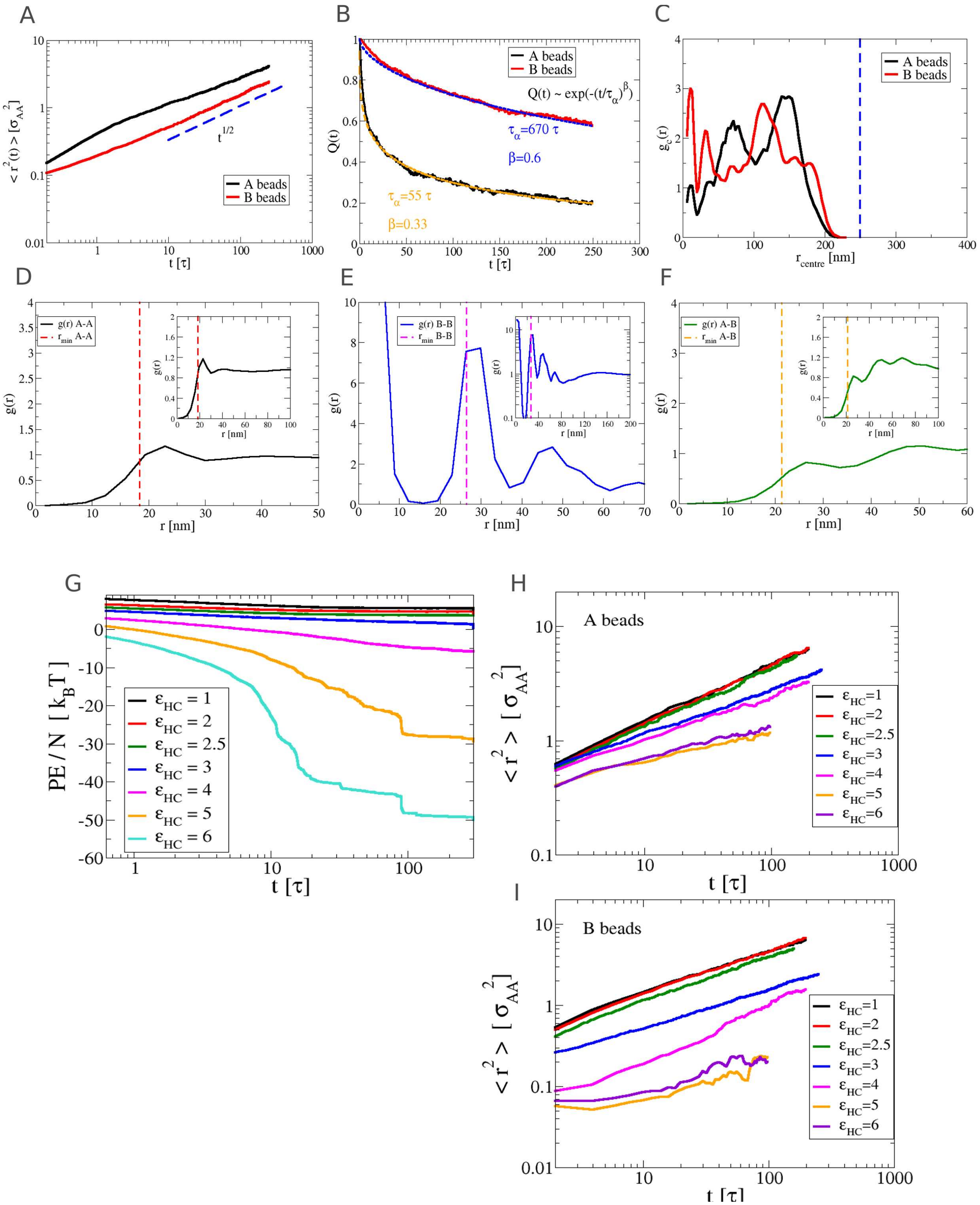

FIG. S6: Mean squared displacement in simulations corresponding to control conditions (**A**), overlap functions (**B**), and relative density distributions of EC / A and HC / B beads as a function of distance from the center of the cavity (**C**). Bead-bead pair correlation functions are shown relative to background density in (**D**) for EC-EC / $A-A$ separations, (**E**) for HC-HC / $B-B$ separations, and (**F**) for EC-HC / $A-B$ separations. (**G**) shows the potential energy per particle (PE/N) for simulations with different HC-HC / $B-B$ affinity, ϵ_{HC}. The mean squared displacements of EC / A beads (**H**) and HC / B beads (**I**) are shown for different values of ϵ_{HC}.

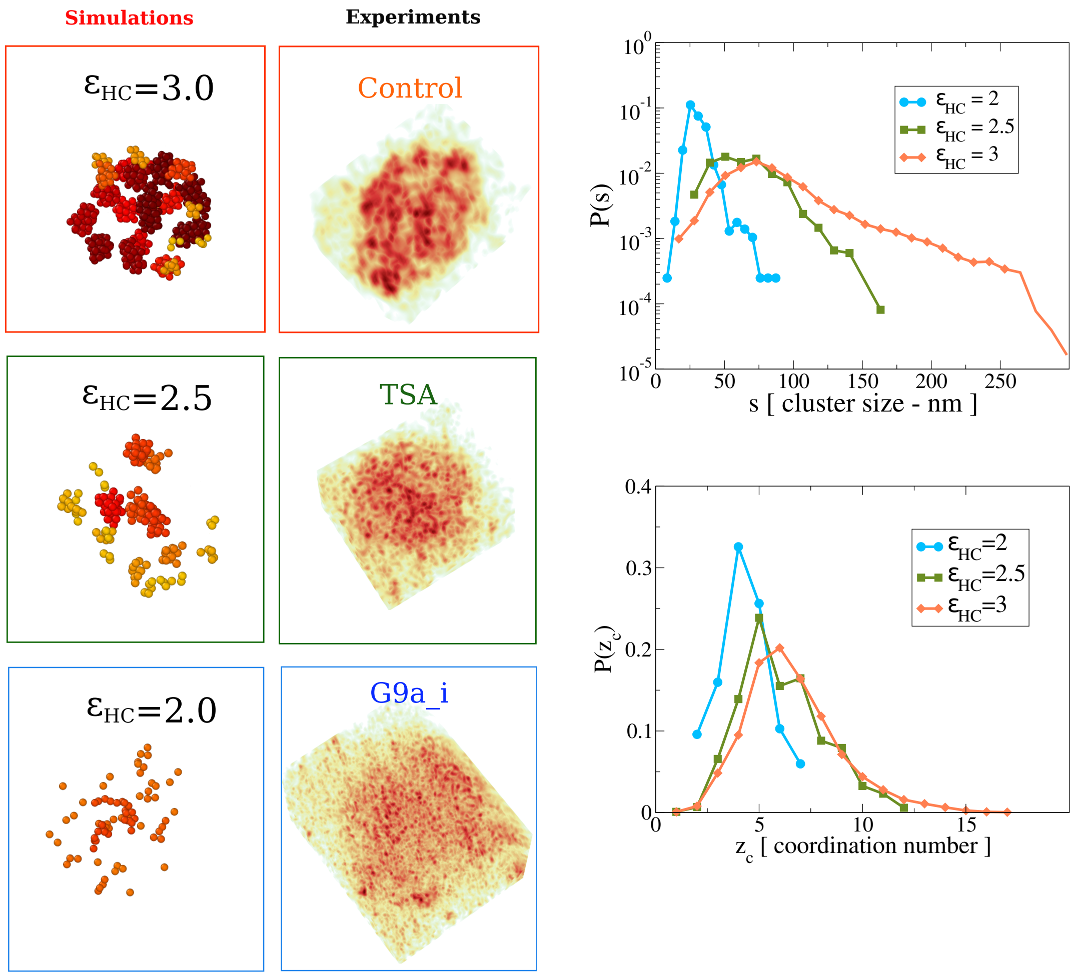

FIG. S7: The cluster size distribution, $P(s)$, and the coordination number distribution, $P(z_c)$, are shown for simulations at different HC-HC / $B-B$ heterochromatin affinities, ϵ_{HC}. Representative images of heterochromatin mesoscale domains from simulations and from segmented CLXT are shown corresponding to each of these conditions. The warmer color represents dense domains in SXT Experiments and larger domains in simulations, whereas the cooler colors represent less dense and smaller domains respectively.

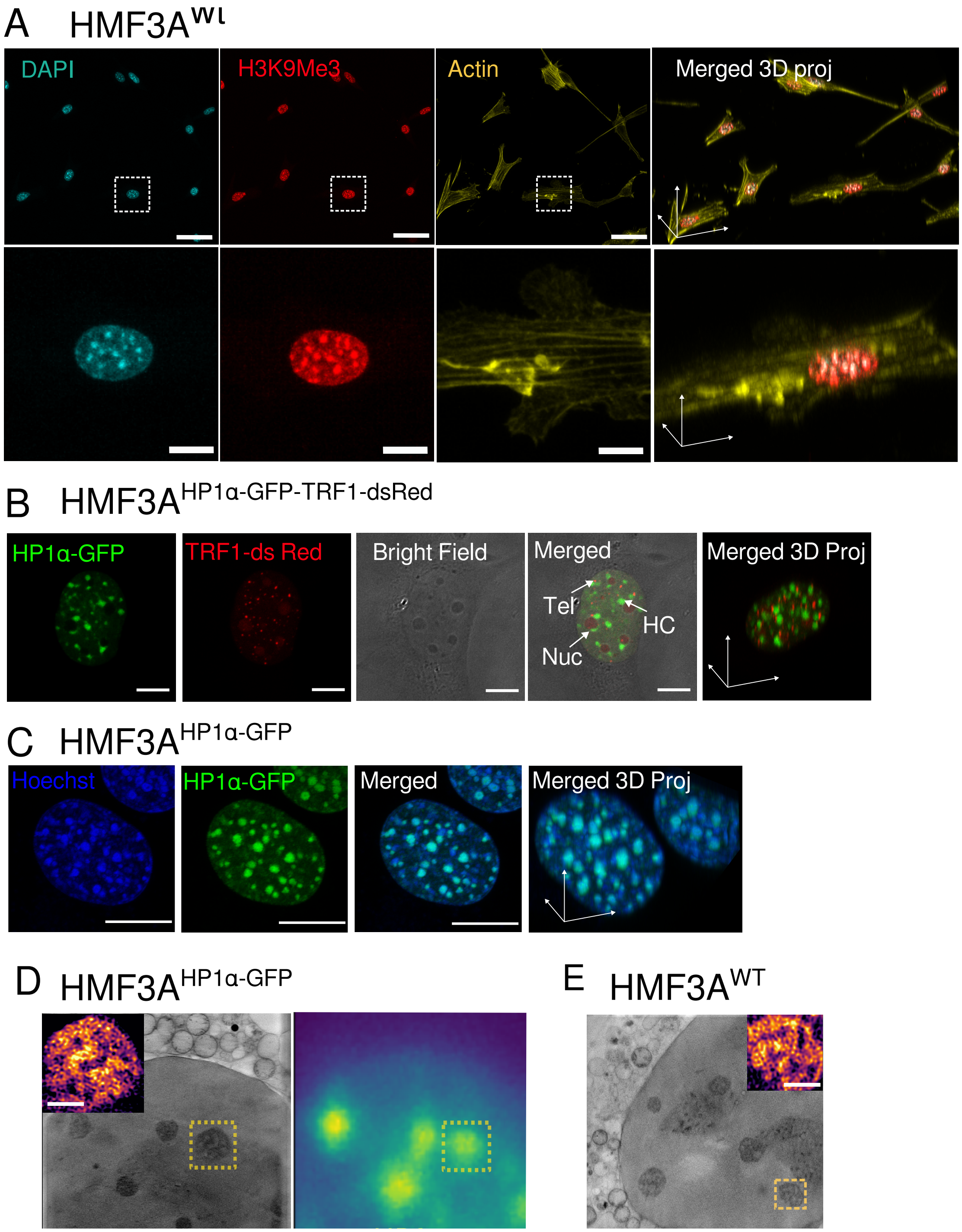

FIG. S8: (**A**) Immunofluorescent images, 3D Projection and corresponding zoomed view of HMF3A^{WT} cells labelled with DAPI, H3K9Me3 and Actin. (**B**) Live cell fluorescent and bright field images of HMF3A$^{HP1\alpha-GFP-TRF1-dsRed}$ showing localization of heterochromatin labeled with HP1α-GFP, telomeric regions labeled with TRF1-dsRed and bright field images showing nucleolus, corresponding merged view and merged 3D projection. (**C**) HMF3A$^{HP1\alpha-GFP}$ cells labeled with Hoechst showing chromatin organization, Heterochromatin regions labeled with HP1α-GFP, corresponding merged view and Merged 3D projection. (**D**) Virtual Z slice of Cryo-SXT and corresponding visible light microscopy of HMF3A expressing HP1α-GFP showing characteristic spatially connected mesoscale domains. (**E**) Virtual Z slice from FOV of subnuclear Cryo-SXTs in HMF3A^{WT}, Inset: Yellow Regions of Interest (ROI) are zoomed in and color-mapped with a local LAC range, arrows indicating the characteristic connected clusters of dense domains in both cells. Inset: 6μm Scale bar(A) 50μm, 10μm, (B) 5μm, (C) 10μm, (D) & (E) 2 μm,

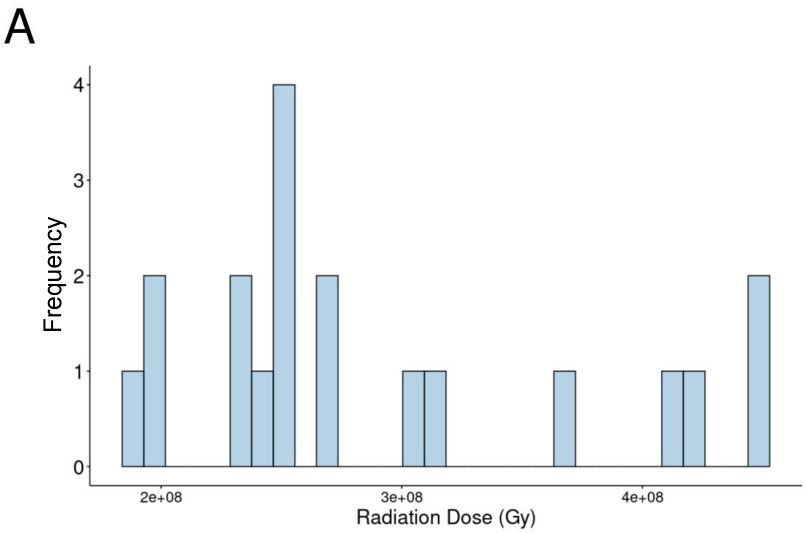

FIG. S9: Maximum possible radiation dose plotted on the x-axis for the cryo SXT data presented in this study.

Supplementary Movie Legends:

Movie S10: Sequential Orthoslices in X and Z direction of the full cell from control condition.

Movie S11-S14: Sequential Orthoslices in X and Z direction of the heterochromatin region from control condition.

Movie S15-S18: Sequential Orthoslices in X and Z direction of the heterochromatin region from TSA treated cell.

Movie S19-S20: Sequential Orthoslices in X and Z direction of the heterochromatin region from G9a inhibitor treated cell.

Movie S21-S22: 60 min live cell video of HMF3A cells overexpressing GFP-HP1α and dsRed-TRF1, showing heterochromatin enriched nucleolar boundaries in 3D axis and merged with bright field respectively.

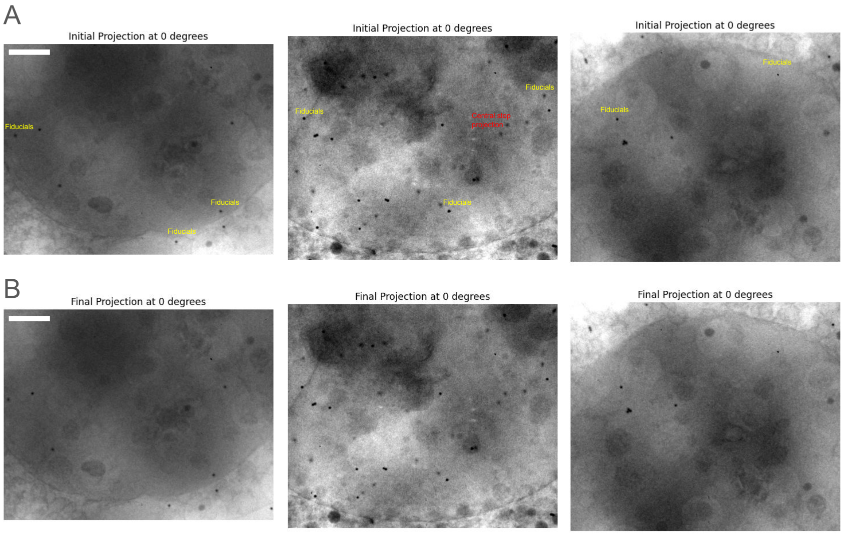

FIG. S23: TXM projection of the subnuclear fields of view (FOV) at 0 degrees (A) before and (B) after tilt series acquisition on which SXT is performed. Yellow annotations label some of the fiducials in these projections, Red annotation shows the artifact due to the central stop. Scale Bar: (A)&(B) 2µm

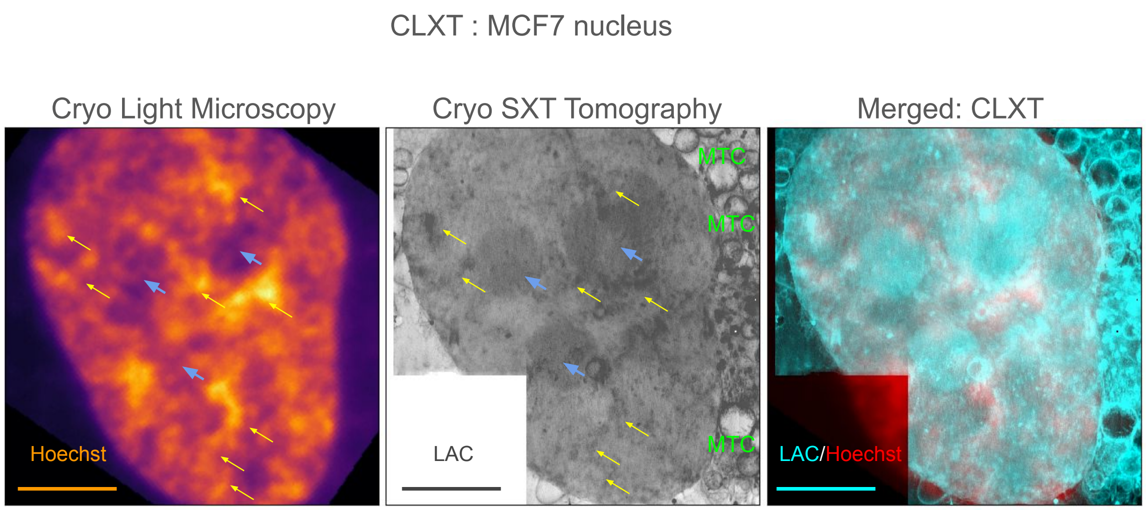

FIG. S24: (A) Cryo Light Microscopy of MCFA cells stained Hoechst. Yellow color and purple background correspond to bright and dark intensity respectively. (B) Virtual Slice of cryo Soft X-ray tomography on the same cells. The yellow arrows, blue arrows, and green text annotate heterochro- matin regions, Nucleoli, and mitochondria respectively. (C) The overlayed CLXT as RGB, with the red channel as the cryo light microscopy image, and the cryo SXT virtual slice as the green and the blue channel. Scale bar (A),(B),& (C) 5µm

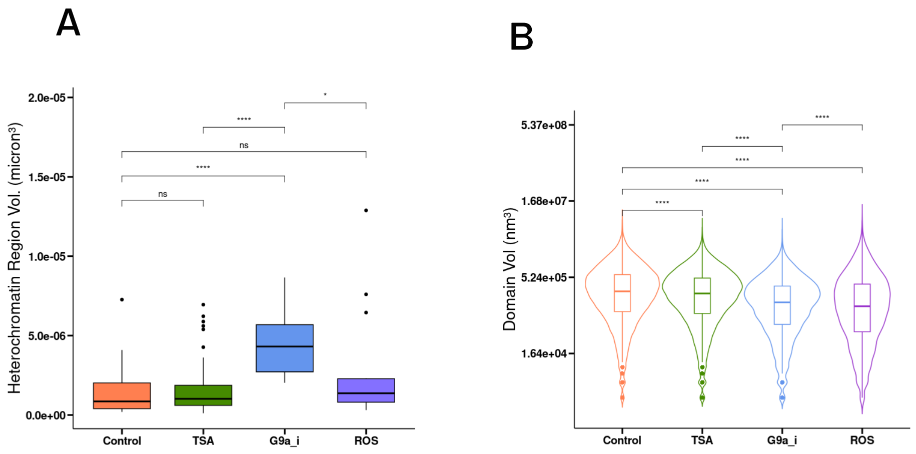

FIG. S25: (A) Volume of heterochromatin regions and (B) Volume of mesoscale domains in HMF3A^{WT} cells under different perturbation conditions
